# Crossfire: the emotional cost of Military Police work

**DOI:** 10.1590/1980-220X-REEUSP-2025-0171en

**Published:** 2026-01-26

**Authors:** Rejane de Fátima Parada Viegas, Karla Gualberto Silva, Sheila Nascimento Pereira de Farias, Elisabete Pimenta Araújo Paz, Katerine Moraes dos Santos, Regina Célia Gollner Zeitoune

**Affiliations:** 1 UNIGAMA – Centro Universitário Gama e Souza, Rio de Janeiro, Brazil.; 2 Universidade Federal do Rio de Janeiro, Escola de Enfermagem Anna Nery, Rio de Janeiro, RJ, Brazil.; 3 Universidade Federal do Rio de Janeiro, Escola de Enfermagem Anna Nery, Departamento de Enfermagem e Saúde Pública, Rio de Janeiro, RJ, Brazil.; 4 Universidade Federal Fluminense, Escola de Enfermagem Aurora de Afonso Costa, Departamento de Fundamentos de Enfermagem e Administração, Niterói, RJ, Brazil.

**Keywords:** Occupational Health, Police, Nursing, Psychological Distress

## Abstract

**Objective:**

To analyze the affective cost of work for military police officers in the metropolitan region of Rio de Janeiro.

**Method:**

Cross-sectional study carried out with 446 military police officers, of both sexes, from 5 military police battalions in Rio de Janeiro. A sociodemographic, work and health conditions questionnaire and the Human Cost at Work Assessment Scale were used. Data were organized, processed, and analyzed using the software *Statistical Package for the Social Sciences*, version 13.1.

**Results:**

The affective cost of work was considered serious to critical among military police officers, with a statistically significant association being observed between the assessment of serious affective cost and medical leave due to bipolar disorder (p = 0.035), cognitive changes in perception (p = 0.009), attention (p = 0.053) and memory (p = 0.015), age (p < 0.001), working time (p < 0.001), and sleep (p < 0.034).

**Conclusion:**

It is essential to promote a healthy environment, raise awareness about the importance of mental health, and seek professional help when necessary. Ensuring police officers’ mental health is critical to their happiness and quality of life, and to their ability to perform their duties effectively. This has positive consequences for society, underscoring the urgent need for ongoing interventions and support in this area.

## INTRODUCTION

The World Health Organization (WHO) and the International Labour Organization (ILO) have issued an urgent call for the adoption of effective measures to address concerns related to worker´s mental health, given the significant impact these issues have on the workplace. According to the WHO World Mental Health Report, published in 2022, in 2019 around 1 billion people lived with mental disorders and 15% of adult workers were affected by psychological problems that impaired their job performance^(1)^.

The work environment of military police officers imposes a high emotional burden, marked by rigid organizational demands, a hierarchical structure, and constant exposure to risky situations, which favors psychological suffering and emotional illness. These conditions can be compared to those faced by public health professionals, who also deal with work overload, fear, feelings of helplessness, and emotional exhaustion. These factors, common to both contexts, highlight the urgent need for institutional policies that promote continuous psychological support, healthy work environments, and leaders capable of dealing with the emotional impacts of work^(2)^.

Although institutional guidelines recommend effective practices to meet workers’ needs, especially regarding mental health, the topic is still treated as taboo in many workplaces. These guidelines direct embracing and caring actions that aim to promote employees’ psychological well-being, but they are often not put into practice. The silence surrounding mental disorders reveals both the stigma that associates suffering with weakness and the difficulty institutions have in implementing effective support actions. Furthermore, management models focused on productivity, unprepared leadership, and fear of reprisals from workers make it difficult to address the problem. In this context, it is essential to promote an organizational culture based on care, with access to psychological support, management training, healthy work practices, and reintegration programs that respect the workers’ limits. Such measures are essential to break the cycle of invisibility that still marks the relationship between mental health and work and represent effective ways of meeting workers’ needs in a humane, respectful, and preventive manner^(3)^.

In the work context of the military police officer, also known as a public security agent, this professional deals with unique demands in their routine, structured by organizational challenges that can trigger various types of illness, with direct impacts on mental health. Evidence shows that these workers have a low overall quality of life, with a strong association between poor work capacity and worsening physical and mental health. Furthermore, factors such as excessive alcohol consumption and younger age are related to poorer mental health, revealing the vulnerability of this occupational group. These findings reflect how the stressful nature of police work, marked by violence, constant risks, and long working hours, contributes to the deterioration of health and reinforces the need for strategies to promote quality of life and prevent illness^(4)^.

The demands that place police officers under constant subjective tension stand out: the need to maintain emotional control in high-risk situations, without legitimate space for expressing weaknesses; the role of society protector, which contrasts with the continuous exposure to violence and threats to one’s own life; the hierarchical rigidity of the military structure, which coexists with the requirement for autonomous decisions in the field; and the demand for institutional loyalty, in the face of the recurring feeling of helplessness when the professional becomes ill. Added to this is the pressure for operational results, often in contexts of scarcity of human and material resources. These structured and persistent contradictions create an organizational scenario that, far from providing support, often contributes to psychological suffering and the chronicity of mental illness among public security agents^(5)^.

Considering this perspective, the high emotional burden involved in police activity stands out, in which the affective cost represents the emotional expenditure resulting from affective responses, feelings and moods. This aspect of the human cost at work is particularly intense in professions with hierarchical organizational structures, as is the case with the Military Police of the State of Rio de Janeiro (PMERJ)^(6)^.

Supporting this statement, a study found that work can negatively affect worker´s mental health, highlighting chronic stress, symptoms of anxiety and depression, a constant feeling of vulnerability, difficulty accessing specialized mental health services, sleep disorders, persistent fatigue, and a high risk of burnout. Such effects are directly associated with emotional overload and the perception of helplessness on the part of institutions^(7)^. Despite the extensive literature available on aspects of mental health among military police officers, there are still gaps in knowledge in this field, especially regarding individual and work-related factors related to illness, as well as protective factors and possible interventions^(8)^.

Considering the complexity of military activity highlighted in the literature^(9)^ and aiming to identify gaps in the human cost of military police work, bibliographic searches were carried out in the Virtual Health Library (VHL) and National Library of Medicine (Pubmed/NLM), of articles published between 2016 and 2021, in English and Portuguese, using the keyword *custo humano* (human cost) and the descriptors *policial, saúde do trabalhador, condições de trabalho, riscos ocupacionais e desgaste profissional* (police officer, worker health, working conditions, occupational risks and professional burnout); and mesh terms police and occupational diseases. Thirty-six articles were selected for analysis, among the 251 publications identified; however, no research addressed the work context of military police officers in Rio de Janeiro and the associated human cost, although some studies address the mental health of this group of workers. Three articles in the Nursing field focused on police officers; however, none of them used the Work and Illness Risk Inventory (ITRA), which was employed in the present study, warranting the present research that aims to fill this identified gap.

Given this scenario, and considering the context that involves the emotional commitment of military police officers in their work environment, the study aimed to analyze the affective cost of work for military police officers in the metropolitan region of Rio de Janeiro.

## METHOD

### Design of Study

This is a cross-sectional study, based on the guidelines of the *Strengthening the Reporting of Observational Studies in Epidemiology* (STROBE) for writing the manuscript.

### Local

The research was carried out in the Military Police Battalions (BPM) that had the greatest armed confrontations in Metropolitan Region I of the state of Rio de Janeiro, according to the high crime rates presented at the National Symposium on Police Victimization in the State of Rio de Janeiro, held by the Military Police in 2019.

### Population and Selection Criteria

The number of participants was determined taking into account the total number of 44.336 military police officers in the state of Rio de Janeiro. The selection criteria were to be military police officers, of both sexes, in the following hierarchical positions: Enlisted personnel (soldiers, corporals, sergeants, and warrant officers) and officers (lieutenants, captains, majors, lieutenant colonels, and colonels), distributed among 5 BPMs in the state of Rio de Janeiro.

All police officers from the selected military units who were present during data collection were considered for participation, provided they met the criteria for fitness A (no health restrictions) and fitness B (with some health restrictions and no restrictions on carrying a weapon) and were able to answer the questions in the questionnaires. Inactive military personnel, on vacation or with aptitude C (psychological problems or other more serious health problems, with restrictions on carrying weapons for a certain period of time) were excluded.

### Sample Definition

For the sample calculation, the EPI-INFO software version 7.2.2.16 was used, with a margin of error of 5.0%, confidence level of 95.0%, proportion estimate of 50.0%, and loss rate of 20.0%. The stratified sample was n = 417. However, other police officers expressed interest in participating in the study during data collection and were included in the sample as they met the inclusion criteria. A total of 446 military police officers participated in the study.

The battalions were named A, B, C, D and E to ensure anonymity, and the participants were distributed by battalion as follows: battalions A (n = 108); B (n = 104); C (n = 98); D (n = 54), and E (n = 82).

### Data Collection Instrument

The collection instrument consisted of a sociodemographic questionnaire and the Human Cost at Work Assessment Scale (EACHT). The questionnaire with questions about the sociodemographic, health and work profile had the following variables: sex, age, having children, hierarchical position, unit of activity, length of service in the military institution, pre-existing health conditions, sick leave and number of hours of sleep. There was no pilot testing of this instrument.

The EACHT is part of ITRA, an inventory validated in Brazil in 2003, adapted and validated again in 2004 and 2006, and subsequently published in 2007. Factor analysis revealed data adequacy, with Kaiser-Meyer-Olkin (KMO) = 0.91, indicating excellent sampling adequacy for applying the model (values above 0.80 are considered very good). An eigenvalue greater than 2 confirms that the extracted factor explains a significant amount of the variance of the items, being higher than the usual cut-off point of 1.0. The factorial solution presented a total explained variance of 44.98%, with 50% of the correlations between items above 0.30, which reinforces the consistency of the factorial structure.

The EACHT consists of five factors, which analyze the demands related to the human cost at work: physical cost (questions 1 to 10); cognitive cost (questions 11 to 20); and affective cost (questions 21 to 30)^(10)^. To meet the objective of this article, an excerpt from the first author’s doctoral thesis was made, and the factor evaluating the affective cost of work^(11)^ was analyzed.

This is a five-point Likert-type scale, which indicates the degree of demand of the job, being: 1 – not at all demanded; 2 – little demanded; 3 – more or less demanded; 4 – quite demanded; and 5 – totally demanded. An individual analysis of each item of the EACHT affective cost factor was carried out using the arithmetic mean and standard deviation. The risk of illness was assessed according to the authors’ guidelines, considering the following averages: above 3.70 – more negative assessment, serious risk of illness; between 3.69 and 2.30 – moderate assessment, critical risk of illness; and below 2.29 – more positive assessment, satisfactory^(10)^.

### Data Collection

Initially, contact was established with the General Commander of the Military Police through the Strategic Affairs Coordination – CAEs, to explain the research proposal and request authorization to carry it out in each unit. After the presentation of the official approval document issued by CAEs, the research was submitted to the Ethics Committee of the proposing institution and, after its approval, data collection began in the different units. Thus, the military personnel were invited to participate in the research and informed about its objectives and the importance of collaboration.

Data collection took place from December 2021 to June 2022, following a schedule previously agreed with participants, in a location defined by respondents. Participants completed the instruments and a deadline was set for their return during the next shift. When the return did not happen within the expected timeframe, a new attempt was made, resulting in the return and no losses.

### Data Analysis and Treatment

To structure and organize the database, the Statistical Package for the Social Sciences (SPSS), version 13.1 for the Windows platform and Excel 365 were used with the double entry method, aiming to reduce possible errors and inconsistencies. All analyses were conducted with a 95% reliability level, with only valid responses being considered.

Data were presented in tables, with their respective absolute and relative frequencies. The quantitative variables (sociodemographic and labor characteristics and the effective cost of EACHT work) were presented using measures of centrality and dispersion. To verify the statistically significant relationship, the Chi-Square Test and Fisher’s Exact Test were used, and to assess the normality of the data, the non-parametric Kolmogorov-Smirnov test was chosen for the numerical variables.

To evaluate the difference between three or more sets of data that were not related, we chose to use the Kruskal-Wallis statistical test.

### Ethical Aspects

The study was approved by the Ethics Committee of the proposing institution following the guidelines of Resolution No. 466/2012 and approved by opinion number 5,137,132. All those involved read the Free and Informed Consent Form (FICF), which was signed, confirming participation in the research, and a copy signed by the researcher was given to each participant. It is worth noting that participation in the research was voluntary, the consent could be withdrawn at any time, and participants could refuse to respond to any of the questions, if they did not feel comfortable. Therefore, the “n” of each variable showed variation in the total sample.

## RESULTS

A total of 446 military police officers from the State of Rio de Janeiro participated in the research. However, some questions on the form were not completed. Due to this loss of information, the “n” differs according to the variables. Of the total number of participants, 86.7% (379) were male and 13.3% (58) were female; regarding marital status, 70.8% (311) were married or living in a common-law marriage, while 29.2% (128) were separated, divorced, widowed, or single. Regarding the number of children, 64.7% (289) reported having them; of these, the majority (47.4%, 137) had only 1 child.

Military police officers were questioned about the reasons for medical leave. In this question, there was the possibility of marking more than one diagnosis; therefore, data are presented in absolute values, based on the number of participants, with the respective diagnoses: anxiety attack (n = 81), depression (n = 64), panic syndrome (n = 21), and bipolar disorder (n = 17).

When asked if they had ever thought about suicide, 15% (66) of participants responded affirmatively. It is worth noting that seven participants did not respond to the question. Regarding cognitive changes, 193 police officers responded affirmatively, with 110 stating that they had presented memory changes, 63 changes in attention, and 20 changes in perception.

Table 1 presents the mean, standard deviation and risk classification of the items that make up the affective cost factor of EACHT’s work.

**Table 1:** Items of the affective cost of work factor of the Human Cost at Work Scale according to mean, standard deviation and risk classification for military police officers of the State of Rio de Janeiro (n = 446) – Rio de Janeiro, Brazil, 2023.

Affective Cost	Mean ± SD*	Risk classification
Having control over emotions	4.06 ± 1.15	Severe
Having an emotional cost	3.67 ± 1.20	Critical
Being forced to deal with aggression from others	3.65 ± 1.33	Critical
Disguising feelings	3.48 ± 1.36	Critical
Having to deal with contradictory orders	3.39 ± 1.17	Critical
Being forced to take care of one's physical appearance	3.23 ± 1.32	Critical
Being forced to be in a good mood	2.54 ± 1.32	Critical
Being nice to others	2.49 ± 1.30	Critical
Transgressing ethical values	2.22 ± 1.50	Satisfactory
Being subjected to constraints	2.17 ± 1.34	Satisfactory
Being forced to praise people	2.08 ± 1.25	Satisfactory
Being forced to smile	1.86 ± 1.20	Satisfactory

*Standard deviation.

Items are presented in descending order from averages. The most negative responses were in relation to “Having control over emotions” (μ = 4.06 ± 1.15), indicating a serious risk of illness; and “Having an emotional cost” (μ = 3.67 ± 1.20), with a critical risk of illness. The most positive response was in relation to “Being forced to smile” (μ = 1.86 ± 1.20), indicating that the police officers believe that they are not forced to smile, which is a positive factor in the work context. The standard deviation was greater than 1.00 in all items of the factor, indicating variability in the responses.

Table 2 presents the association between the affective cost factor and the variables: ever thought about suicide, reason for medical leave, cognitive changes, age, time in PMERJ, and hours of sleep per day. The variables that, according to the literature, can influence the relationship between the work context and the human cost at work were chosen. Among the variables analyzed, a statistically significant association was observed between the assessment of severe affective cost and medical leave due to “bipolar disorder”; and cognitive changes related to “Perception” and “Memory”. Statistically significant differences were also identified in the three numerical variables “Age”, “Time in PMERJ” and “Hours of sleep per day”, indicating that these variables were associated with the levels of affective cost of work among military police officers.

**Table 2:** Association between the affective cost factor and sociodemographic, work and health condition (n = 446) variables – Rio de Janeiro, Brazil, 2023.

	EACHT – Affective Cost			
Variables	Severe n (%)	Critical n (%)	Satisfactory n (%)	p-value
**Have you ever thought about suicide?**				
Yes	26 (40.0)	25 (38.5)	14 (21.5)	0.568**
No	122 (33.6)	162 (44.6)	79 (21.8)
**Reason for Medical Leave**				
Depression	25 (39.1)	22 (34.3)	17 (26.6)	0.252**
Anxiety attack	31 (38.3)	33 (40.7)	17 (21.0)	0.706**
Bipolar disorder	**9 (56.2)**	2 (12.5)	5 (31.3)	**0.035** **
**Cognitive changes**				
Perception	**13 (65.0)**	6 (30.0)	1 (5.0)	**0.009** **
Attention	29 (46.0)	26 (41.3)	8 (12.7)	0.053**
Memory	**48 (43.6)**	47 (42.8)	15 (13.6)	**0.015** **
	**Median (P_25_; P_75_)**	**Median (P_25_; P_75_)**	**Median (P_25_; P_75_)**
Age	37.0 (33.0; 41.0)	37.0 (33.0; 42.0)	41,0 (36.0; 46.0)	**< 0.001^A^**
Time in PMERJ**	9.0 (3.0; 14.5)	9.0 (3.0; 18.3)	12.5 (8.0; 21.3)	**< 0.001^A^**
Hours of sleep per day	6.0 (5.5; 7.0)	6,0 (6.0; 7.0)	7.0 (6.0; 8.0)	**0.034^A^**

*Chi-square.

**Military Police of the State of Rio de Janeiro.

^A^Kuskal-Wallis.

## DISCUSSION

The results of this dissertation corroborate previous studies that indicate the predominance of male military police officers, aged between 30 and 40, married or in a common-law marriage, and with one to two children. This demographic profile highlights the importance of the marital and family context in understanding the impacts of professional activity on these workers’ personal lives^(11,12)^.

In this regard, such characteristics directly influence reproductive decisions, which have revealed a tendency towards a reduction in the number of children among military police officers, a phenomenon often associated with the specificities of the profession. Furthermore, the increase in mortality among police officers in the state of Rio de Janeiro, with a growth of 45.5% in 2021, totaling 64 deaths, intensifies the fear of leaving children orphaned, negatively impacting family planning^(13)^.

These aspects, combined with the high emotional cost of the profession, manifest themselves in symptoms such as anxiety, fear and depression, compromising both the mental health and interpersonal relationships of these professionals^(14)^.

The work environment has a significant influence on professionals’ motivation and the risk of illness, as evidenced in studies with different job categories, such as military police, health and education professionals. Excessive workloads, inadequate conditions, and a lack of adequate training are common factors that contribute to physical and mental health problems, although the intensity and impact of these factors vary depending on the sector in which they operate. Mental and emotional overload can result from long working hours, a near-total lack of rest breaks, high levels of responsibility, technical and material limitations, high demands, and often low pay. These conditions, combined with organizational pressures, can negatively impact the relationship between professionals and users and increase the risk of illness, requiring careful analysis of the specificities of each work environment to develop effective prevention and health promotion strategies^(15,16,17)^.

In this research, the main reasons for sick leave reflect the psychological suffering of the category and have pathologies as consequences. In this case, the capacity of the military work organization to weaken individuals and lead to psychological decompensations, when the worker has exhausted all psycho-affective and intellectual resources to respond to work demands^(18)^. A reality not restricted to military police officers, data from Brazil’s Social Security confirms a 29% increase in the granting of social security sickness benefits in 2019 and 2020, with an emphasis on illnesses related to mental and behavioral disorders, which are the third largest cause of work leaves due to impairment in Brazil^(19)^.

Specifically in relation to the work of the military police officer, direct or indirect contact with violent events that result in death or threat thereof, serious injury and sexual assault causes traumatic experiences for those involved, which, in turn, can trigger various psycho-emotional illnesses and other conditions, especially post-traumatic stress disorder (PTSD), depression, substance abuse, social problems, feelings of frustration with work, and increased demand for medical services^(20)^.

The affective cost at work is characterized by the emotional demand in response to affective reactions, feelings, and mood^(10)^. In the assessment of the military police officers in this study, the emotional cost of the work they perform poses a critical risk of illness, with the most negatively evaluated items being having control over emotions, having an emotional cost, and being forced to deal with the aggression of others, the first being evaluated as serious. In the context of the work of military police officers, it is clear that, in addition to facing varied demands in the workplace, violence is part of their daily lives, being one of the reasons why there is a great manifestation of emotions in the performance of their duties.

Amidst this chaotic context, the acceptance of violence in cities is perceived through a repetitive process, which manifests itself in policing practices that have been ingrained over the years. Furthermore, it is important to emphasize the importance of approaching the problem from the perspective of fear, which plays a fundamental role in the increase in violence^(21)^. In this logic, the public security agent occupies a position as a causal link in State violence, inverting his real professional performance based on what may arise from his (re)action based on a violent attitude arising from fear, caused by the adversity of the social context, such as working conditions, training, psychological monitoring, and adaptation of working hours.

Abstention in this scenario implies a serious danger to society. This is because ignoring it opens the door for a police officer with psychological problems to, during tense moments, commit abuse and act aggressively. In fact, mental disorders weaken these professionals’ reasoning skills, as they affect their ability to use resources that enable them to find solutions that do not involve aggression and violence present in the daily routine of the profession^(22)^.

Still from the perspective of the emotional cost at work, police work on the streets involves dealing with various emotional demands, such as interacting with offenders, criminals, victims, and the community in general, which requires the ability to express different feelings. Professional identity is essential in this scenario, allowing the police officer to adopt strategies to express emotions according to the situation.

However, it is important to highlight that police officers deal daily with different audiences, each with specific emotional needs, making emotional control even more challenging, as they must demonstrate empathy and solidarity to victims, authority and firmness to suspects and criminals, and professionalism when dealing with the general population^(23)^. This leads us to believe that one of the most important and challenging responsibilities of the police is to keep violence under control without being affected by it.

Therefore, the symbolic, practical and political effects must be considered both in the social sphere and in the daily life of Public Security professionals, who need to develop both physical and psychological strategies to deal with the reality of their activity, which involves violence directed at themselves and others^(20)^. This activity interferes with private life; military rigor and exposure to violence shape the way people relate to each other. This influence goes beyond relationships, guiding the professional’s character, thus demonstrating the dimension that the profession can have in the police officer’s life^(24)^.

The lack of institutional support in addressing workplace violence, combined with the imminent risk of death and growing organizational pressures, creates a scenario conducive to psychological suffering among military police officers. This condition, combined with physical exhaustion and emotional vulnerability, can trigger inappropriate behavioral responses during critical situations and high-risk operations^(25)^.

In this sense, a study carried out with military police officers from Minas Gerais revealed that 14.8% of participants presented significant symptoms of depression and burnout. These emotional illnesses were strongly associated with factors such as occupational stress, job dissatisfaction, negative perception of mental health, and low resilience, highlighting the negative impact of working conditions and institutional and social pressures on the mental health of these professionals^(26)^.

Despite not having a statistically significant relationship, 15% of police officers have already considered suicide. In this regard, research carried out at a military police institution in southern Brazil identified 14 cases between 2012 and 2016^(27)^. The widespread fatigue experienced daily in the professional environment of police officers is the main factor that leads to both mental and physical deterioration, and can lead to suicide as a way of putting an end to their own torment^(28)^.

In agreement with these results, an integrative review that sought to identify the most relevant themes related to police officers’ mental health in the literature, from 2012 to 2018, allowed contextualizing studies on this topic. It became clear that, despite being conducted in different countries, the research points to stress, PTSD, depression, anxiety, burnout and suicide as the main health problems studied^(29)^. In this regard, PTSD is listed as one of the main causes that lead to suicide, being explained by the constant exposure to traumatic events while fulfilling one’s obligations^(30)^.

Seeking help from specialized professionals is crucial to identify signs and factors that may indicate mental disorders, avoiding drastic actions or denying the existence of the problem. The lack of mental health support is alarming, especially given changes in social relationships, which can increase pressure and trigger crises^(31)^.

Although military police officers face routines of high exposure to risks, violence, and intense stress, constant access to weapons reinforces an institutional culture of invulnerability, in which expressing psychological fragility poses a threat to one’s career. This situation intensifies occupational stress and makes it difficult to communicate psychiatric symptoms—many choose to hide them for fear of reputational damage, retaliation, or lack of confidentiality. Hierarchical rigidity and the absence of institutional spaces for dialogue aggravate this denial of emotional suffering, reducing the use of mental health services and preventing effective access to treatment, perpetuating a cycle of illness that goes undiagnosed or formally untreated^(32)^.

The daily lives of these professionals, according to an integrative review published in 2024, show that the police profession is one of the most stressful, with a high incidence of work-related mental illnesses^(25)^. Factors such as rigid hierarchy, lack of time off, irregular shifts, operational pressure, and chronic emotional demands make these workers vulnerable to mental illness^(32)^. Studies also show that absences due to psychiatric disorders (such as depression, anxiety and post-traumatic stress) are recurrent, reinforcing the importance of institutional care policies^(25)^. These elements highlight the urgency of interventions capable of breaking the cycle of emotional silencing that impairs these professionals’ mental health.

The results indicate that the affective cost faced by military police officers is associated with emotional illness, including mood disorders, cognitive changes related to perception and memory, and sleep difficulties. The work environment, especially when the barracks are located in areas with a high incidence of armed conflict, imposes intense emotional demands and contributes to the development of these clinical conditions. Such conditions significantly affect professionals’ mental health, resulting in sick leave due to disorders such as bipolar disorder, cognitive impairment, and variations related to age and length of service^(25)^.

In this study, it was found that younger police officers with less time in service reported the affective cost of work as severe or critical, indicating greater psychosocial vulnerability. These results differ from those observed in another study, carried out with military police officers from Sergipe, which associated poor sleep quality and impaired quality of life, especially among younger professionals, with a median age of 40 and up to seven years of service^(33)^. This work identified impacts on the biological, psychological, relational, and economic dimensions, revealing that sleep deprivation substantially compromises these professionals’ mental health. The discrepancies between the studies may reflect regional and operational particularities of the corporations, reinforcing the importance of considering such variables when analyzing illness in police work.

Although working conditions in the military police service are demanding and often exhausting, certain components of the affective cost of work are perceived less negatively. In a comprehensive analysis, items such as not feeling obligated to smile or compliment people were rated with the lowest averages, indicating that these emotional demands are seen as less burdensome. Additionally, many police officers report a strong sense of belonging to the force, institutional pride, and satisfaction in performing punitive tasks on behalf of the State, which suggests a positive emotional dimension even in the face of adversity and ongoing stress^(6)^. These findings align with this study and show that, even in environments with high levels of occupational stress, many military police officers present medium to high levels of work engagement, with emphasis on enthusiasm, dedication and pride in the metric function, which reinforces that certain affective aspects of work can generate emotional resilience rather than significant suffering.

The results of this research confirm that performing external tasks during patrols and direct confrontation with crime are significant indicators of emotional exhaustion among police officers. These activities expose officers to high levels of violence and risk, contributing to considerable stress. Furthermore, these roles are often performed under precarious working conditions, exacerbating the negative impact on police officer well-being.

Emotional problems in this work environment are alarming and have become more frequent. Factors such as stress, pressure to achieve results, fierce competition, and a work-life imbalance may be contributing to the emergence of emotional problems, including those identified in this research, such as anxiety, depression, and bipolar disorder.

As a limitation of the research, the type of cross-sectional study that does not allow for statements of cause and effect between the variables investigated can be considered. Therefore, it is suggested that longitudinal studies be designed to understand the phenomenon over time.

The study contributed to reflections on the importance of preventing mental health problems in the workplace, requiring collaboration between organizations and workers. It is essential to promote a healthy environment, raise awareness about the importance of mental health, and seek professional help when necessary. Ensuring police officers’ mental health is critical to their happiness and quality of life, corroborating the urgent need for ongoing interventions and support in this area.

## CONCLUSION

The analysis of the affective cost of the work of military police officers in the metropolitan region of Rio de Janeiro obtained a critical evaluation for the majority of the factors presented, with the most negative response, that with an assessment of serious risk for illness, being that related to having control of emotions, followed by having an emotional cost, with a critical risk for illness. The association analysis indicated that the assessment of severe affective cost was related to medical leave due to bipolar disorder and cognitive alterations in perception and memory. There was also an association between age, length of service in the Military Police and hours of sleep, indicating that younger police officers, with less time on the job and fewer hours of sleep, presented a severe assessment of the affective cost at work. Another fact worth highlighting is that 15% of police officers have considered committing suicide. The nature of the military police officer’s work exposes them to emotional distress, affective problems, and mood swings, which can result in psychiatric problems and cognitive changes, leading to illness, absences, and even suicidal thoughts.

## Data Availability

The entire dataset supporting the results of this study is available upon request to the corresponding author.
