# Supplementary material for: Crossfire: the emotional cost of Military Police work
Source: Rev Esc Enferm USP. 2026 Jan 26;60:e20250171. doi: 10.1590/1980-220X-REEUSP-2025-0171en (PMC12858051; doi:10.1590/1980-220X-REEUSP-2025-0171en)
Supplement: Translation

# Translation: Fogo cruzado: custo afetivo do trabalho de Policiais Militares

**DOI:** 10.1590/1980-220X-REEUSP-2025-0171pt

**Authors:** Rejane de Fátima Parada Viegas, Karla Gualberto Silva, Sheila Nascimento Pereira de Farias, Elisabete Pimenta Araújo Paz, Katerine Moraes dos Santos, Regina Célia Gollner Zeitoune

**Affiliations:** 1 UNIGAMA – Centro Universitário Gama e Souza, Rio de Janeiro, Brasil.; 2 Universidade Federal do Rio de Janeiro, Escola de Enfermagem Anna Nery, Rio de Janeiro, RJ, Brasil.; 3 Universidade Federal do Rio de Janeiro, Escola de Enfermagem Anna Nery, Departamento de Enfermagem e Saúde Pública, Rio de Janeiro, RJ, Brasil.; 4 Universidade Federal Fluminense, Escola de Enfermagem Aurora de Afonso Costa, Departamento de Fundamentos de Enfermagem e Administração, Niterói, RJ, Brasil.

**Keywords:** Saúde ocupacional, Polícia, Enfermagem, Angústia Psicológica

## Abstract

**Objetivo:**

Analisar o custo afetivo do trabalho em policiais militares da região metropolitana do Rio de Janeiro.

**Método:**

Estudo transversal, realizado com 446 policiais militares, de ambos os sexos, de 5 batalhões da polícia militar do Rio de Janeiro. Utilizou-se um questionário sociodemográfico, laboral e de condições de saúde e a Escala de Avaliação do Custo Humano no Trabalho. Os dados foram organizados, processados e analisados com o auxílio do Programa *Statistical Package for the Social Sciences*, versão 13.1.

**Resultados:**

O custo afetivo do trabalho foi considerado de grave a crítico entre os policiais militares, sendo observada associação estatisticamente significativa entre a avaliação do custo afetivo grave e licença médica em virtude de transtorno bipolar (p = 0,035), alterações cognitivas de percepção (p = 0,009), atenção (p = 0,053) e memória (p = 0,015), idade (p < 0,001), tempo de trabalho (p < 0,001) e sono (p < 0,034).

**Conclusão:**

É essencial promover um ambiente saudável, conscientizar sobre a importância da saúde mental e buscar ajuda profissional quando necessário. Garantir a saúde mental dos policiais é fundamental para sua felicidade e qualidade de vida, e bom desempenho da sua função, com consequências positivas para a sociedade, corroborando a necessidade urgente de intervenções e suportes contínuos nesta área.

## INTRODUÇÃO

A Organização Mundial da Saúde (OMS) e a Organização Internacional do Trabalho (OIT) lançaram um apelo urgente pela adoção de medidas efetivas que enfrentem as preocupações relacionadas à saúde mental dos trabalhadores, diante do impacto expressivo que essas questões exercem sobre o ambiente laboral. Segundo o Relatório Mundial de Saúde Mental da OMS, publicado em 2022, em 2019 cerca de 1 bilhão de pessoas viviam com transtornos mentais e 15% dos trabalhadores adultos foram afetados por problemas psicológicos que prejudicaram seu desempenho no emprego^(1)^.

O ambiente de trabalho dos policiais militares impõe uma elevada carga emocional, marcada por exigências organizacionais rígidas, estrutura hierarquizada e constante exposição a situações de risco, o que favorece o sofrimento psíquico e o adoecimento emocional. Tais condições podem ser comparadas às enfrentadas por profissionais da saúde pública, que também convivem com sobrecarga de trabalho, medo, sensação de desamparo e exaustão emocional. Esses fatores, comuns a ambos os contextos, evidenciam a necessidade urgente de políticas institucionais que promovam suporte psicológico contínuo, ambientes de trabalho saudáveis e lideranças capacitadas para lidar com os impactos emocionais do trabalho^(2)^.

Embora as diretrizes institucionais recomendem práticas eficazes para atender às necessidades dos trabalhadores, sobretudo no que se refere à saúde mental, o tema ainda é tratado como tabu em muitos ambientes laborais. Essas diretrizes orientam ações de acolhimento e cuidado que visam promover o bem-estar psicológico dos colaboradores, mas muitas vezes não são colocadas em prática. O silêncio que cerca os transtornos psíquicos revela tanto o estigma que associa sofrimento à fraqueza quanto a dificuldade das instituições em implementar ações efetivas de acolhimento. Além disso, modelos de gestão centrados na produtividade, o despreparo das lideranças e o receio de represálias por parte dos trabalhadores dificultam o enfrentamento do problema. Nesse contexto, é fundamental promover uma cultura organizacional baseada no cuidado, com acesso a apoio psicológico, capacitação de gestores, práticas laborais saudáveis e programas de reintegração que respeitem os limites dos trabalhadores. Tais medidas são essenciais para romper o ciclo de invisibilidade que ainda marca a relação entre saúde mental e trabalho e representam formas eficazes de atender às necessidades dos trabalhadores de maneira humana, respeitosa e preventiva^(3)^.

No contexto laboral do policial militar, também denominado agente de segurança pública, esse profissional lida com demandas únicas em sua rotina, estruturadas por desafios organizacionais que podem desencadear diversos tipos de adoecimento, com reflexos diretos na saúde mental. Evidências demonstram que esses trabalhadores apresentam baixa qualidade de vida geral, com forte associação entre capacidade laboral precária e piora dos componentes físicos e mentais da saúde. Além disso, fatores como consumo excessivo de álcool e idade mais jovem estão relacionados a uma menor saúde mental, revelando a vulnerabilidade desse grupo ocupacional. Tais achados refletem como a natureza estressante da atividade policial, marcada pela violência, riscos constantes e longas jornadas, contribui para a deterioração da saúde e reforça a necessidade de estratégias de promoção da qualidade de vida e de prevenção do adoecimento^(4)^.

Destacam-se exigências que colocam os policiais em constante tensão subjetiva: a necessidade de manter controle emocional em situações de alto risco, sem que haja espaço legítimo para expressão de fragilidades; o papel de protetor da sociedade, que se contrapõe à exposição contínua à violência e à ameaça à própria vida; a rigidez hierárquica da estrutura militar, que convive com a exigência de decisões autônomas em campo; e a cobrança por lealdade institucional, frente à sensação recorrente de desamparo quando o profissional adoece. Soma-se a isso a pressão por resultados operacionais, muitas vezes em contextos de escassez de recursos humanos e materiais. Essas contradições, estruturadas e persistentes, configuram um cenário organizacional que, longe de oferecer suporte, frequentemente contribui para o sofrimento psíquico e a cronicidade do adoecimento mental entre os agentes de segurança pública^(5)^.

Considerando essa perspectiva, destaca-se a elevada carga emocional envolvida na atividade policial, em que o custo afetivo representa o dispêndio emocional decorrente de respostas afetivas, sentimentos e estados de humor. Esse aspecto do custo humano no trabalho é particularmente intenso em profissões com estruturas organizacionais hierarquizadas, como é o caso da Polícia Militar do Estado do Rio de Janeiro (PMERJ)^(6)^.

Corroborando essa afirmativa, um estudo identificou que o trabalho pode afetar negativamente a saúde mental dos trabalhadores, evidenciando quadros de estresse crônico, sintomas de ansiedade e depressão, sensação constante de vulnerabilidade, dificuldades de acesso a serviços especializados em saúde mental, distúrbios do sono, fadiga persistente e elevado risco de burnout. Tais efeitos estão diretamente associados à sobrecarga emocional e à percepção de desamparo por parte das instituições^(7)^. Apesar da ampla literatura disponível sobre os aspectos da saúde mental de policiais militares, ainda se observam lacunas de conhecimento nesse campo, especialmente no que se refere aos fatores individuais e laborais relacionados ao adoecimento, bem como aos fatores protetivos e às intervenções possíveis^(8)^.

Considerando a complexidade da atividade militar apontada na literatura^(9)^ e visando identificar lacunas sobre o custo humano no trabalho de policiais militares, foram realizadas buscas bibliográficas na Biblioteca Virtual em Saúde (BVS) e National Library of Medicine (Pubmed/NLM), de artigos publicados no período de 2016 a 2021, em inglês e português, a partir da palavra-chave custo humano e dos descritores policial, saúde do trabalhador, condições de trabalho, riscos ocupacionais e desgaste profissional; e *mesh terms police* e *occupational diseases*. Foram selecionados para análise 36 artigos, dentre as 251 publicações identificadas, porém, nenhuma pesquisa abordou o contexto de trabalho de policiais militares no Rio de Janeiro e o custo humano associado, apesar de alguns estudos tratarem da saúde mental deste grupo de trabalhadores. Três artigos da área de Enfermagem focaram em policiais; entretanto, nenhum deles utilizou o Inventário do Trabalho e Riscos de Adoecimento (ITRA), que foi empregado no presente estudo, o que justifica a realização da presente pesquisa que visa preencher esta lacuna identificada.

Diante desse cenário, e considerando o contexto que envolve o comprometimento emocional do policial militar em seu ambiente de trabalho, o estudo teve como objetivo analisar o custo afetivo do trabalho em policiais militares da região metropolitana do Rio de Janeiro.

## MÉTODO

### Desenho do Estudo

Trata-se de um estudo de corte transversal, embasado nas diretrizes do *Strengthening the Reporting of Observational Studies in Epidemiology* (STROBE) para a redação do manuscrito.

### Local do Estudo

A pesquisa foi realizada nos Batalhões da Polícia Militar (BPM) que apresentavam os maiores enfrentamentos armados na Região Metropolitana I do estado do Rio de Janeiro, de acordo com os altos índices de criminalidade apresentados no Simpósio Nacional de Vitimização Policial no Estado do Rio de Janeiro, realizado pela Polícia Militar em 2019.

### População e Critérios de Seleção

O número de participantes foi determinado levando em consideração a quantidade total de 44.336 de policiais militares no estado do Rio de Janeiro. Foram critérios de seleção ser policial militar, de ambos os sexos, das posições hierárquicas: Praças (soldados, cabos, sargentos e subtenentes) e oficiais (tenentes, capitães, majores, tenentes-coronéis e coronéis), distribuídos entre 5 BPM do estado do Rio de Janeiro.

Todos os policiais das unidades militares selecionadas que estavam presentes durante a coleta de dados foram considerados para participação, desde que atendessem aos critérios de aptidão A (sem restrições de saúde) e aptidão B (com algumas restrições de saúde e sem restrição ao porte de arma) e estivessem aptos a responder às perguntas dos questionários. Militares inativos, de férias ou com aptidão C (problemas de ordem psíquica ou outro problema de saúde mais sério, com restrição ao porte de arma por tempo determinado), foram excluídos.

### Definição da Amostra

Para o cálculo amostral, empregou-se o software EPI-INFO na sua versão 7.2.2.16, com margem de erro de 5,0%, nível de confiança de 95,0%, estimativa de proporção de 50,0% e taxa de perda de 20,0%. A amostra estratificada foi de n = 417. No entanto, outros policiais manifestaram interesse em participar do estudo durante a coleta de dados e foram incluídos na amostra, pois atendiam os critérios de inclusão. Participaram do estudo 446 policiais militares.

Os batalhões foram denominados de A, B, C, D e E para garantir o anonimato, e os participantes foram assim distribuídos por batalhão: A (n = 108); B (n = 104); C (n = 98); D (n = 54) e batalhão E (n = 82).

### Instrumento de Coleta de Dados

O instrumento de coleta foi composto por um questionário sociodemográfico e a Escala de Avaliação do Custo Humano no Trabalho (EACHT). O questionário com perguntas sobre o perfil sociodemográfico, de saúde e trabalho tinha as seguintes variáveis: sexo, idade, possuir filhos, posição hierárquica, unidade de atuação, tempo de serviço na instituição militar, condições de saúde pré-existentes, licença com afastamento médico e quantidade de horas de sono. Não houve teste piloto deste instrumento.

A EACHT integra o ITRA, inventário validado no Brasil em 2003, adaptado e novamente validado em 2004 e 2006, sendo posteriormente publicado em 2007. A análise fatorial revelou adequação dos dados, com Kaiser-Meyer-Olkin (KMO) = 0,91, indicando excelente adequação amostral para a aplicação do modelo (valores acima de 0,80 são considerados muito bons). O eigenvalue (autovalor) maior que 2 confirma que o fator extraído explica uma quantidade significativa da variância dos itens, sendo superior ao ponto de corte usual de 1,0. A solução fatorial apresentou variância total explicada de 44,98%, com 50% das correlações entre os itens acima de 0,30, o que reforça a consistência da estrutura fatorial.

A EACHT é composta por cinco fatores, que analisam as demandas relacionadas ao custo humano no trabalho: custo físico (questões de 1 a 10); custo cognitivo (questões de 11 a 20); e custo afetivo (questões de 21 a 30)^(10)^. Para atender ao objetivo deste artigo, realizou-se um recorte da tese de doutorado da primeira autora, sendo analisado o fator que avalia o custo afetivo do trabalho^(11)^.

Trata-se de uma escala do tipo Likert de cinco pontos, que indica o grau de exigência do trabalho, sendo: 1 – nada exigido; 2 – pouco exigido; 3 – mais ou menos exigido; 4 – bastante exigido; e 5 – totalmente exigido. Foi realizada a análise individual de cada item do fator custo afetivo da EACHT por meio da média aritmética e desvio padrão. O risco de adoecimento foi avaliado conforme a orientação dos autores, considerando-se as seguintes médias: acima de 3,70 – avaliação mais negativa, risco grave de adoecimento; entre 3,69 e 2,30 – avaliação moderada, risco crítico de adoecimento; e abaixo de 2,29 – avaliação mais positiva, satisfatória^(10)^.

### Coleta de Dados

Inicialmente, estabeleceu-se contato com o Comandante Geral da Polícia Militar por meio da Coordenação de Assuntos Estratégicos – CAEs, a fim de explicar a proposta da pesquisa e solicitar autorização para realizá-la em cada unidade. A partir da apresentação do documento oficial de aprovação emitido pela CAEs, a pesquisa foi submetida ao Comitê de Ética da instituição proponente e, após sua aprovação, deu-se início à coleta de dados nas diferentes unidades. Desta forma, os militares foram convidados a participar da pesquisa e informados sobre seus objetivos e a importância da colaboração.

A coleta de dados ocorreu de dezembro de 2021 a junho de 2022, seguindo um cronograma previamente combinado com os participantes, em local definido pelos respondentes. Os participantes preencheram os instrumentos e foi estabelecido o prazo para a devolução no próximo plantão. Quando a devolução não aconteceu dentro do prazo previsto, uma nova tentativa foi realizada, havendo a devolução e não havendo perdas.

### Análise e Tratamento dos Dados

Para a estruturação e organização do banco de dados, foram empregados os programas Statistical Package for the Social Sciences (SPSS), versão 13.1. para a plataforma Windows e o Excel 365 com o método de dupla digitação, visando reduzir possíveis equívocos e incongruências. Todas as análises foram conduzidas com um nível de confiabilidade de 95%, sendo consideradas apenas as respostas válidas.

Os dados foram apresentados em tabelas, com suas respectivas frequências absolutas e relativas. As variáveis quantitativas (características sociodemográficas e laborais e as do custo efetivo no trabalho da EACHT) foram apresentadas pelas medidas de centralidade e dispersão. Para verificar a relação estatisticamente significativa foram utilizados o Teste Qui-Quadrado e o Teste Exato de Fisher e, para avaliar a normalidade dos dados, optou-se pelo teste não paramétrico de Kolmogorov-Smirnov nas variáveis numéricas.

Com o intuito de avaliar a diferença entre três ou mais conjuntos de dados que não estavam relacionados, optou-se por utilizar o teste estatístico de Kruskal-Wallis.

### Aspectos Éticos

O estudo foi aprovado pelo Comitê de Ética da instituição proponente seguindo as diretrizes da Resolução n.º 466/2012 e aprovado pelo parecer número 5.137.132. Todos os envolvidos leram o Termo de Consentimento Livre e Esclarecido (TCLE), o qual foi assinado, confirmando a participação na pesquisa, e uma via assinada pelo pesquisador foi entregue a cada participante. Cabe ressaltar que a participação na pesquisa foi de livre e espontânea vontade, sendo permitida retirada do consentimento a qualquer momento, assim como a não resposta a qualquer uma das questões, caso o participante não se sentisse à vontade. Portanto, o “n” de cada variável apresentou variação do total amostral.

## RESULTADOS

Participaram da pesquisa 446 policiais militares do Estado do Rio de Janeiro. No entanto, algumas questões do formulário não foram preenchidas. Em virtude dessa perda de informações, o “n” difere de acordo com as variáveis. Do total de participantes 86,7% (379) eram do sexo masculino e 13,3% (58) do sexo feminino; quanto ao estado civil 70,8% (311) eram casados(as) ou viviam em união, enquanto 29,2% (128) eram separados, divorciados, viúvos ou solteiros. Com relação ao número de filhos, 64,7% (289) referiram tê-los; destes, a maioria (47,4%, 137) tinha apenas 1 filho.

Os policiais militares foram questionados quanto aos motivos de licenças médicas. Nesta questão, havia a possibilidade de marcar mais de um diagnóstico, portanto, os dados estão apresentados em valores absolutos, a partir do número de participantes, com os respectivos diagnósticos: crise de ansiedade (n = 81), depressão (n = 64), síndrome do pânico (n = 21) e transtorno bipolar (n = 17).

Quando questionados se alguma vez já pensaram em suicídio, 15% (66) dos participantes responderam afirmativamente. Vale destacar que sete participantes não responderam ao questionamento. No que diz respeito às alterações cognitivas, 193 policiais responderam afirmativamente, sendo que 110 afirmaram ter apresentado alterações de memória, 63 alterações na atenção e 20 alterações de percepção.

A Tabela 1 apresenta média, desvio padrão e classificação de risco dos itens que compõem o fator custo afetivo do trabalho da EACHT.

**Tabela 1:** Itens do fator custo afetivo do trabalho da Escala de Custo Humano do Trabalho segundo média, desvio padrão e classificação de risco para policiais militares do Estado Rio de Janeiro (n = 446) – Rio de Janeiro, Brasil, 2023.

Custo Afetivo	Média ± DP*	Classificação de risco
Ter controle das emoções	4,06 ± 1,15	Grave
Ter custo emocional	3,67 ± 1,20	Crítico
Ser obrigado a lidar com agressividade dos outros	3,65 ± 1,33	Crítico
Disfarçar os sentimentos	3,48 ± 1,36	Crítico
Ter que lidar com ordens contraditórias	3,39 ± 1,17	Crítico
Ser obrigado a cuidar da aparência física	3,23 ± 1,32	Crítico
Ser obrigado a ter bom humor	2,54 ± 1,32	Crítico
Ser bonzinho com os outros	2,49 ± 1,30	Crítico
Transgredir valores éticos	2,22 ± 1,50	Satisfatório
Ser submetido a constrangimentos	2,17 ± 1,34	Satisfatório
Ser obrigado a elogiar as pessoas	2,08 ± 1,25	Satisfatório
Ser obrigado a sorrir	1,86 ± 1,20	Satisfatório

*Desvio padrão.

Os itens estão apresentados em ordem decrescente a partir das médias. As respostas mais negativas foram em relação a “Ter controle das emoções” (μ = 4,06 ± 1,15), indicando risco grave para o adoecimento; e “Ter custo emocional” (μ = 3,67 ± 1,20), com risco crítico para o adoecimento. A resposta mais positiva foi em relação a “Ser obrigado a sorrir” (μ = 1,86 ± 1,20), indicando que os policiais avaliam que não são obrigados a sorrir, sendo este um fator positivo no contexto de trabalho. O desvio padrão foi maior que 1,00 em todas os itens do fator, indicando variabilidade nas respostas.

A Tabela 2 apresenta a associação entre o fator custo afetivo e as variáveis: já pensou em suicídio, motivo de licença médica, alterações cognitivas, idade, tempo na PMERJ e horas de sono por dia. Elegeram-se as variáveis que, de acordo com a literatura, podem influenciar a relação entre o contexto de trabalho e o custo humano no trabalho. Dentre as variáveis analisadas, foi observada associação estatisticamente significativa entre a avaliação do custo afetivo grave e licença médica em virtude de “transtorno bipolar”; e alterações cognitivas relacionados à “Percepção” e “Memória”. Foram também identificadas diferenças estatisticamente significativas nas três variáveis numéricas “Idade”, “Tempo na PMERJ” e “Horas de sono por dia” indicando que essas variáveis estavam associadas com os níveis de custo afetivo do trabalho entre os policiais militares.

**Tabela 2:** Associação entre o fator custo afetivo e variáveis sociodemográficas, laborais e condições de saúde (n = 446) – Rio de Janeiro, Brasil, 2023.

	EACHT – Custo Afetivo			
Variáveis	Grave n (%)	Crítico n (%)	Satisfatório n (%)	p-valor
**Já pensou alguma vez em suicídio**				
Sim	26 (40,0)	25 (38,5)	14 (21,5)	0,568**
Não	122 (33,6)	162 (44,6)	79 (21,8)
**Motivo da Licença Médicas**				
Depressão	25 (39,1)	22 (34,3)	17 (26,6)	0,252**
Crise de ansiedade	31 (38,3)	33 (40,7)	17 (21,0)	0,706**
Transtorno bipolar	**9 (56,2)**	2 (12,5)	5 (31,3)	0,035**
**Alterações cognitivas**				
Percepção	**13 (65,0)**	6 (30,0)	1 (5,0)	0,009**
Atenção	29 (46,0)	26 (41,3)	8 (12,7)	0,053**
Memória	**48 (43,6)**	47 (42,8)	15 (13,6)	0,015**
	**Mediana (P_25_; P_75_)**	**Mediana (P_25_; P_75_)**	**Mediana (P_25_; P_75_)**
Idade	37,0 (33,0; 41,0)	37,0 (33,0; 42,0)	41,0 (36,0; 46,0)	**< 0,001^A^**
Tempo na PMERJ**	9,0 (3,0; 14,5)	9,0 (3,0; 18,3)	12,5 (8,0; 21,3)	**< 0,001^A^**
Horas de sono por dia	6,0 (5,5; 7,0)	6,0 (6,0; 7,0)	7,0 (6,0; 8,0)	**0,034^A^**

*Qui-quadrado.

**Polícia Militar do Estado do Rio de Janeiro.

^A^AKuskal-Wallis

## DISCUSSÃO

Os resultados desta tese corroboram estudos anteriores que indicam o predomínio de policiais militares do sexo masculino, com idade entre 30 e 40 anos, casados ou em união estável, e com um a dois filhos. Esse perfil demográfico evidencia a importância do contexto conjugal e familiar para a compreensão dos impactos da atividade profissional sobre a vida pessoal desses trabalhadores^(11,12)^.

Nesse sentido, tais características influenciam diretamente as decisões reprodutivas, as quais têm revelado uma tendênciade redução no número de filhos entre policiais militares, fenômeno frequentemente associado às especificidades da profissão. Ademais, o aumento da mortalidade entre policiais no estado do Rio de Janeiro, com um crescimento de 45,5% em 2021, totalizando 64 óbitos, intensifica o temor de deixar filhos órfãos, impactando negativamente o planejamento familiar^(13)^.

Esses aspectos, somados ao elevado custo afetivo da profissão, manifestam-se em sintomas como ansiedade, medo e depressão, comprometendo tanto a saúde mental quanto as relações interpessoais desses profissionais^(14)^.

O ambiente de trabalho exerce influência significativa na motivação dos profissionais e no risco de adoecimento, conforme evidenciado em estudos com diferentes categorias laborais, como policiais militares, profissionais da saúde e educação. A carga horária excessiva, condições inadequadas e a ausência de treinamento adequado são fatores comuns que contribuem para problemas de saúde física e mental, ainda que a intensidade e a repercussão desses fatores variem conforme o setor de atuação. A sobrecarga mental e emocional pode advir de jornadas prolongadas, falta quase total de pausas para descanso, alta responsabilidade, limitações técnicas e materiais, elevada demanda e, frequentemente, baixa remuneração. Essas condições, aliadas às pressões organizacionais, podem gerar um impacto negativo na relação dos profissionais com os usuários e aumentar o risco de adoecimento, demandando uma análise cuidadosa das especificidades de cada ambiente laboral para o desenvolvimento de estratégias eficazes de prevenção e promoção da saúde^(15-17)^.

Nesta pesquisa, os principais motivos de licença médica traduzem o sofrimento psíquico da categoria e têm como consequências as patologias. Neste caso, a capacidade da organização do trabalho militar de fragilizar os indivíduos e propiciar descompensações psicológicas, quando o trabalhador esgotou todos os recursos de origem psicoafetiva e intelectual para responder às demandas de trabalho^(18)^. Realidade não restrita aos policiais militares, dados da Previdência Social do Brasil afirmam um aumento de 29%, nos anos 2019 e 2020, na concessão de auxílio-doença previdenciário, com destaque para as doenças relacionadas às desordens mentais e comportamentais, que são a terceira maior causa de afastamentos laborais incapacitantes no Brasil^(19)^.

Especificamente em relação ao trabalho do policial militar, o contato direto ou indireto com eventos violentos que resultam em morte ou ameaça dela, lesão grave e violação sexual acarreta vivências traumáticas para os envolvidos, que, por sua vez, podem desencadear diversos adoecimentos psicoemocionais e outras condições, sobretudo transtorno de estresse pós-traumático (TEPT), depressão, abuso de substâncias, problemas sociais, sentimento de frustração com o trabalho e aumento da procura por serviços médicos^(20)^.

O custo afetivo no trabalho caracteriza-se pela demanda emocional em resposta a reações afetivas, sentimentos e estado de humor^(10)^. Na avaliação dos policiais militares deste estudo, o custo afetivo do trabalho realizado por eles oferece um risco crítico de adoecimento, destacando-se entre os itens mais negativamente avaliados ter controle das emoções, ter custo emocional e ser obrigado a lidar com a agressividade dos outros, sendo o primeiro avaliado como grave. No contexto de trabalho dos policiais militares, é evidente que, além de enfrentarem diversas demandas no ambiente de trabalho, a violência faz parte do seu cotidiano, sendo um dos motivos pelos quais há uma grande manifestação de emoções na realização de suas atribuições.

Em meio a esse contexto caótico, a aceitação da violência nas cidades é percebida por meio de um processo repetitivo, que se manifesta nas práticas policiais enraizadas ao longo dos anos. Além disso, é importante ressaltar a importância de abordar o problema a partir do sentimento de medo, que desempenha um papel fundamental no aumento da violência^(21)^. Nessa lógica, o agente de segurança pública ocupa uma posição de elo causador da violência do Estado, invertendo-se o seu real desempenho profissional em função do que poderá advir de sua (re)ação pautada numa atitude violenta oriunda do temor, ocasionado pela adversidade do contexto social, como as condições de trabalho, treinamento, acompanhamento psicológico e adequação da carga horária.

Abster-se diante desse cenário implica em um sério perigo para a sociedade. Isso ocorre porque ignorá-lo é abrir espaço para que um policial com problemas psicológicos possa, durante momentos de tensão, cometer abusos e agir de forma agressiva. De fato, o distúrbio mental enfraquece a habilidade de raciocínio desses profissionais, já que afeta a capacidade de utilizar recursos que possibilitam encontrar soluções que não envolvam agressão e violência presentes no dia a dia da profissão^(22)^.

Ainda na perspectiva do custo afetivo no trabalho, a atuação policial nas ruas envolve lidar com diversas demandas emocionais, como interagir com infratores, criminosos, vítimas e a comunidade em geral, o que requer habilidade para expressar diferentes sentimentos. A identidade profissional é essencial nesse cenário, permitindo ao policial adotar estratégias para expressar emoções conforme a situação.

No entanto, é importante destacar que os policiais lidam diariamente com públicos diferentes, cada um com necessidades emocionais específicas, tornando o controle emocional ainda mais desafiador, pois devem demonstrar empatia e solidariedade às vítimas, autoridade e firmeza aos suspeitos e criminosos, e profissionalismo ao lidar com a população em geral^(23)^. Isso nos leva a pensar que uma das responsabilidades mais importantes e desafiadoras da polícia é manter a violência sob controle sem se deixar afetar por ela.

Dessa forma, devem-se considerar os efeitos simbólicos, práticos e políticos tanto na esfera social quanto no dia a dia do profissional de Segurança Pública, que precisa desenvolver estratégias tanto físicas quanto psicológicas para lidar com a realidade de sua atividade, que envolve a violência direcionada a si mesmo e aos outros^(20)^. Esta atividade interfere na vida particular, o rigor militar e a exposição à violência moldam a forma de se relacionar. Essa influência vai além dos relacionamentos, passando a nortear o caráter do profissional, dessa forma exibindo-se a dimensão que a profissão pode ter na vida do policial^(24)^.

A ausência de suporte institucional no enfrentamento da violência laboral, associada ao risco iminente de morte e às crescentes pressões organizacionais, configura um cenário propício ao sofrimento psicológico dos policiais militares. Essa condição, somada ao desgaste físico e à vulnerabilidade emocional, pode desencadear respostas comportamentais inadequadas durante situações críticas e operações de alto risco^(25)^.

Nesse sentido, um estudo realizado com policiais militares de Minas Gerais revelou que 14,8% dos participantes apresentaram sintomas significativos de depressão e burnout. Esses quadros de adoecimento emocional estiveram fortemente associados a fatores como estresse ocupacional, insatisfação com o trabalho, percepção negativa da saúde mental e baixa resiliência, evidenciando o impacto negativo das condições laborais e das cobranças institucionais e sociais sobre a saúde mental desses profissionais^(26)^.

Apesar de não ter relação estatística significativa, 15% de policiais já pensaram em suicídio, Nesse aspecto, pesquisa realizada em instituição policial-militar do Sul do Brasil identificou 14 casos entre os anos 2012 e 2016^(27)^. A ampla fadiga experimentada diariamente no ambiente profissional dos policiais é o principal fator que leva à deterioração tanto mental quanto física, podendo levar ao suicídio como uma maneira de pôr fim ao seu próprio tormento^(28)^.

Em concordância com esses resultados, uma revisão integrativa que procurou identificar os temas mais relevantes relacionados à saúde mental dos policiais na literatura, no período de 2012 a 2018, possibilitou contextualizar os estudos sobre esse tema. Ficou claro que, apesar de realizadas em diferentes países, as pesquisas apontam o estresse, o TEPT, a depressão, a ansiedade, o esgotamento profissional e o suicídio como os principais problemas de saúde estudados^(29)^. Nesse aspecto, o TEPT está relacionado como uma das principais causas que levam ao suicídio, sendo explicado pela constante exposição a eventos traumáticos durante o cumprimento de suas obrigações^(30)^.

A busca por ajuda de profissionais especializados é crucial para identificar sinais e fatores que possam indicar transtornos mentais, evitando ações drásticas ou a negação da existência do problema. A falta de suporte em saúde mental é alarmante, especialmente diante das mudanças nas relações sociais, que podem aumentar a pressão e desencadear crises^(31)^.

Embora os policiais militares enfrentem rotinas de alta exposição a riscos, violência e estresse intenso, o acesso constante a armamento reforça uma cultura institucional de invulnerabilidade, na qual expressar fragilidade psicológica representa ameaça à carreira. Essa configuração intensifica o estresse ocupacional e dificulta a comunicação de sintomas psiquiátricos — muitos optam por ocultá-los por medo de prejuízo à reputação, retaliações ou falta de confidencialidade. A rigidez hierárquica e a ausência de espaços institucionais de diálogo agravam essa negação do sofrimento emocional, reduzindo o uso de serviços de saúde mental e impedindo o acesso efetivo a tratamento, perpetuando um ciclo de adoecimento não diagnosticado ou tratado formalmente^(32)^.

O cotidiano desses profissionais, conforme revisão integrativa publicada em 2024, evidencia que a profissão policial é uma das mais estressantes, com alta incidência de doenças psíquicas relacionadas ao trabalho^(25)^. Fatores como hierarquia rígida, ausência de folgas, turno irregular, pressão operacional, e exigências emocionais crônicas tornam esses trabalhadores vulneráveis ao adoecimento mental^(32)^. Estudos também apontam que os afastamentos por transtornos psiquiátricos (como depressão, ansiedade e estresse pós-traumático) são recorrentes, reforçando a relevância de políticas institucionais de cuidado^(25)^. Tais elementos destacam a urgência de intervenções capazes de romper o ciclo de silenciamento emocional que compromete a saúde mental desses profissionais.

Os resultados indicam que o custo afetivo enfrentado pelos policiais militares está associado ao adoecimento emocional, incluindo transtornos do humor, alterações cognitivas relacionadas à percepção e memória, além de dificuldades de sono. O ambiente de trabalho, especialmente quando o quartel está situado em áreas de alta incidência de confrontos armados, impõe demandas emocionais intensas e contribui para o desenvolvimento desses quadros clínicos. Tais condições afetam significativamente a saúde mental dos profissionais, refletindo em licenças médicas por transtornos como o bipolar, prejuízos cognitivos e variações relacionadas à idade e ao tempo de serviço^(25)^.

Nesta pesquisa, verificou-se que policiais mais jovens e com menos tempo de serviço relataram o custo afetivo do trabalho como grave ou crítico, indicando maior vulnerabilidade psicossocial. Esses resultados diferem daqueles observados em outro estudo, realizado com policiais militares de Sergipe, que associou má qualidade do sono e comprometimento da qualidade de vida principalmente a profissionais mais jovens, com mediana de 40 anos e até sete anos de serviço^(33)^. Esse trabalho identificou impactos nas dimensões biológica, psicológica, relacional e econômica, revelando que a privação de sono compromete substancialmente a saúde mental desses profissionais. As divergências entre os estudos podem refletir particularidades regionais e operacionais das corporações, reforçando a importância de considerar tais variáveis ao analisar o adoecimento no trabalho policial.

Embora as condições de trabalho no serviço policial militar sejam exigentes e frequentemente desgastantes, certos componentes do custo afetivo do trabalho são percebidos de modo menos negativo. Em uma análise completa, itens como não se sentirem obrigados a sorrir ou elogiar pessoas foram avaliados com as menores médias, sinalizando que essas demandas emocionais são vistas como menos penosas. Adicionalmente, muitos policiais relatam forte senso de pertencimento à corporação, orgulho institucional e satisfação ao desempenhar tarefas punitivas em nome do Estado, o que sugere uma dimensão emocional positiva mesmo diante de adversidades e estresse contínuo^(6)^. Esses achados alinham-se a este estudo e mostram que, mesmo em ambientes com elevados níveis de estresse ocupacional, muitos policiais militares apresentam níveis médios a altos de engajamento no trabalho, com destaque para entusiasmo, dedicação e orgulho na função métrica, o que reforça que certos aspectos afetivos do trabalho podem gerar resiliência emocional, em vez de sofrimento significativo.

Os resultados desta pesquisa confirmam que a execução de tarefas externas durante as rondas ostensivas e o confronto direto com a criminalidade são indicadores significativos de esgotamento emocional entre os policiais. Essas atividades expõem os policiais a altos níveis de violência e risco, contribuindo para um estresse considerável. Além disso, essas funções são frequentemente realizadas em condições laborais precárias, exacerbando o impacto negativo no bem-estar dos policiais.

Os problemas afetivos neste ambiente de trabalho são alarmantes e têm se tornado mais frequentes. Fatores como estresse, pressão por resultados, competição acirrada e desequilíbrio entre vida profissional e pessoal podem estar contribuindo para o surgimento de problemas emocionais, inclusive os identificados nesta pesquisa, como ansiedade, depressão e transtorno bipolar.

Como limitação da pesquisa, pode ser considerado o tipo de estudo transversal que não permite afirmações de causa e efeito entre as variáveis investigadas. Sendo assim, sugere-se o delineamento de estudos longitudinais para compreensão do fenômeno ao longo do tempo.

O estudo contribuiu para reflexões sobre a importância da prevenção de problemas mentais no local de trabalho exigindo a colaboração entre as organizações e os trabalhadores. É essencial promover um ambiente saudável, conscientizar sobre a importância da saúde mental e buscar ajuda profissional quando necessário. Garantir a saúde mental dos policiais é fundamental para sua felicidade e qualidade de vida, corroborando a necessidade urgente de intervenções e suportes contínuos nesta área.

## CONCLUSÃO

A análise do custo afetivo do trabalho de policiais militares da região metropolitana do Rio de Janeiro obteve avaliação crítica para a maioria dos fatores apresentados, sendo a resposta mais negativa, com avaliação de risco grave para o adoecimento, aquela relativa a ter controle das emoções, seguida de ter custo emocional, com risco crítico para o adoecimento. A análise de associação indicou que a avaliação do custo afetivo grave esteve relacionada à licença médica em virtude de transtorno bipolar e alterações cognitivas de percepção e memória. Também houve associação entre idade, tempo de trabalho na PM e horas de sono, indicando que policiais mais jovens, com menos tempo de trabalho e menos horas de sono apresentaram avaliação grave para o custo afetivo no trabalho. Outro dado que merece destaque é o fato de que 15% dos policiais já pensaram em suicídio. A natureza do trabalho do policial militar expõe esse profissional ao dispêndio emocional, problemas afetivos e alterações de humor, que podem ter como consequências problemas psiquiátricos e alterações cognitivas, levando a adoecimentos, afastamentos e mesmo a pensamentos suicidas.

## Data Availability

Todo o conjunto de dados que dá suporte aos resultados deste estudo está disponível mediante solicitação ao autor correspondente.

# Translation: Fuego cruzado: costo afectivo del trabajo de los policías militares

**DOI:** 10.1590/1980-220X-REEUSP-2025-0171es

**Authors:** Rejane de Fátima Parada Viegas, Karla Gualberto Silva, Sheila Nascimento Pereira de Farias, Elisabete Pimenta Araújo Paz, Katerine Moraes dos Santos, Regina Célia Gollner Zeitoune

**Affiliations:** 1 UNIGAMA – Centro Universitário Gama e Souza, Rio de Janeiro, Brasil.; 2 Universidade Federal do Rio de Janeiro, Escola de Enfermagem Anna Nery, Rio de Janeiro, RJ, Brasil.; 3 Universidade Federal do Rio de Janeiro, Escola de Enfermagem Anna Nery, Departamento de Enfermagem e Saúde Pública, Rio de Janeiro, RJ, Brasil.; 4 Universidade Federal Fluminense, Escola de Enfermagem Aurora de Afonso Costa, Departamento de Fundamentos de Enfermagem e Administração, Niterói, RJ, Brasil.

**Keywords:** Salud Laboral, Policia, Enfermería, Distrés Psicológico

## Abstract

**Objetivo:**

Analizar el costo afectivo del trabajo en los policías militares de la región metropolitana de Río de Janeiro.

**Método:**

Estudio transversal realizado con 446 policías militares, de ambos sexos, de 5 batallones de la policía militar de Río de Janeiro. Se utilizó un cuestionario sociodemográfico, laboral y de condiciones de salud, así como la Escala de Evaluación del Costo Humano en el Trabajo. Los datos se organizaron, procesaron y analizaron con la ayuda del programa Statistical Package for the Social Sciences, versión 13.1.

**Resultados:**

El costo afectivo del trabajo se consideró de grave a crítico entre los policías militares, observándose una asociación estadísticamente significativa entre la evaluación del costo afectivo grave y la baja médica por trastorno bipolar (p = 0,035), alteraciones cognitivas de la percepción (p = 0,009), la atención (p = 0,053) y la memoria (p = 0,015), la edad (p < 0,001), el tiempo de trabajo (p < 0,001) y el sueño (p < 0,034).

**Conclusión:**

Es esencial promover un entorno saludable, concienciar sobre la importancia de la salud mental y buscar ayuda profesional cuando sea necesario. Garantizar la salud mental de los policías es fundamental para su felicidad y calidad de vida, así como para el buen desempeño de su función, con consecuencias positivas para la sociedad, lo que corrobora la urgente necesidad de intervenciones y apoyos continuos en esta área.

## INTRODUCCIÓN

La Organización Mundial de la Salud (OMS) y la Organización Internacional del Trabajo (OIT) han lanzado un llamamiento urgente para que se adopten medidas eficaces que aborden las preocupaciones relacionadas con la salud mental de los trabajadores, dado el impacto significativo que estas cuestiones tienen en el entorno laboral. Según el Informe Mundial sobre Salud Mental de la OMS, publicado en 2022, en 2019 alrededor de mil millones de personas vivían con trastornos mentales y el 15% de los trabajadores adultos se veían afectados por problemas psicológicos que perjudicaban su rendimiento en el trabajo^(1)^.

El entorno de trabajo de los policías militares impone una elevada carga emocional, marcada por exigencias organizativas rígidas, una estructura jerárquica y una exposición constante a situaciones de riesgo, lo que favorece el sufrimiento psíquico y la enfermedad emocional. Estas condiciones pueden compararse con las que enfrentan los profesionales de la salud pública, que también conviven con la sobrecarga de trabajo, el miedo, la sensación de desamparo y el agotamiento emocional. Estos factores, comunes a ambos contextos, ponen de manifiesto la urgente necesidad de políticas institucionales que promuevan el apoyo psicológico continuo, entornos de trabajo saludables y líderes capacitados para lidiar con los impactos emocionales del trabajo^(2)^.

Aunque las directrices institucionales recomiendan prácticas eficaces para atender las necesidades de los trabajadores, especialmente en lo que se refiere a la salud mental, el tema sigue siendo tabú en muchos entornos laborales. Estas directrices orientan las acciones de acogida y atención destinadas a promover el bienestar psicológico de los empleados, pero a menudo no se ponen en práctica. El silencio que rodea a los trastornos psíquicos revela tanto el estigma que asocia el sufrimiento a la debilidad como la dificultad de las instituciones para implementar acciones efectivas de acogida. Además, los modelos de gestión centrados en la productividad, la falta de preparación de los líderes y el temor a represalias por parte de los trabajadores dificultan el enfrentamiento del problema. En este contexto, es fundamental promover una cultura organizacional basada en el cuidado, con acceso a apoyo psicológico, capacitación de gerentes, prácticas laborales saludables y programas de reintegración que respeten los límites de los trabajadores. Estas medidas son esenciales para romper el ciclo de invisibilidad que aún marca la relación entre la salud mental y el trabajo, y representan formas eficaces de atender las necesidades de los trabajadores de manera humana, respetuosa y preventiva^(3)^.

En el contexto laboral del policía militar, también denominado agente de seguridad pública, este profesional se enfrenta a exigencias únicas en su rutina, estructuradas por retos organizativos que pueden desencadenar diversos tipos de enfermedades, con repercusiones directas en la salud mental. Las evidencias demuestran que estos trabajadores presentan una baja calidad de vida general, con una fuerte asociación entre la precaria capacidad laboral y el empeoramiento de los componentes físicos y mentales de la salud. Además, factores como el consumo excesivo de alcohol y la edad más joven están relacionados con una menor salud mental, lo que revela la vulnerabilidad de este grupo ocupacional. Estos hallazgos reflejan cómo la naturaleza estresante de la actividad policial, marcada por la violencia, los riesgos constantes y las largas jornadas, contribuye al deterioro de la salud y refuerza la necesidad de estrategias de promoción de la calidad de vida y prevención de enfermedades^(4)^.

Cabe destacar las exigencias que someten a los policías a una tensión subjetiva constante: la necesidad de mantener el control emocional en situaciones de alto riesgo, sin que haya un espacio legítimo para expresar debilidades; el papel de protector de la sociedad, que se contrapone a la exposición continua a la violencia y a la amenaza a la propia vida; la rigidez jerárquica de la estructura militar, que convive con la exigencia de decisiones autónomas sobre el terreno; y la exigencia de lealtad institucional, frente a la sensación recurrente de desamparo cuando el profesional enferma. A esto se suma la presión por obtener resultados operativos, a menudo en contextos de escasez de recursos humanos y materiales. Estas contradicciones, estructuradas y persistentes, configuran un escenario organizacional que, lejos de ofrecer apoyo, a menudo contribuye al sufrimiento psíquico y a la cronicidad de las enfermedades mentales entre los agentes de seguridad pública^(5)^.

Teniendo en cuenta esta perspectiva, cabe destacar la elevada carga emocional que conlleva la actividad policial, en la que el costo afectivo representa el gasto emocional derivado de las respuestas afectivas, los sentimientos y los estados de ánimo. Este aspecto del costo humano en el trabajo es particularmente intenso en profesiones con estructuras organizativas jerárquicas, como es el caso de la Policía Militar del Estado de Río de Janeiro (PMERJ)^(6)^.

Corroborando esta afirmación, un estudio identificó que el trabajo puede afectar negativamente la salud mental de los trabajadores, evidenciando cuadros de estrés crónico, síntomas de ansiedad y depresión, sensación constante de vulnerabilidad, dificultades para acceder a servicios especializados en salud mental, trastornos del sueño, fatiga persistente y alto riesgo de agotamiento. Estos efectos están directamente asociados a la sobrecarga emocional y a la percepción de desamparo por parte de las instituciones^(7)^. A pesar de la amplia bibliografía disponible sobre los aspectos de la salud mental de los policías militares, aún se observan lagunas de conocimiento en este campo, especialmente en lo que se refiere a los factores individuales y laborales relacionados con la enfermedad, así como a los factores protectores y las posibles intervenciones^(8)^.

Teniendo en cuenta la complejidad de la actividad militar señalada en la literatura^(9)^y con el fin de identificar lagunas sobre el costo humano en el trabajo de los policías militares, se realizaron búsquedas bibliográficas en la Biblioteca Virtual en Salud (BVS) y la Biblioteca Nacional de Medicina (Pubmed/NLM) de artículos publicados entre 2016 y 2021, en inglés y portugués, a partir de la palabra clave costo humano y los descriptores policía, salud del trabajador, condiciones de trabajo, riesgos ocupacionales y desgaste profesional; y mesh terms*police*y*occupational diseases*. Se seleccionaron para su análisis 36 artículos, de entre las 251 publicaciones identificadas, pero ninguna investigación abordó el contexto laboral de los policías militares en Río de Janeiro y el costo humano asociado, a pesar de que algunos estudios trataban la salud mental de este grupo de trabajadores. Tres artículos del área de Enfermería se centraron en los policías; sin embargo, ninguno de ellos utilizó el Inventario de Trabajo y Riesgos de Enfermedad (ITRA), que se empleó en el presente estudio, lo que justifica la realización de la presente investigación, que tiene como objetivo llenar esta laguna identificada.

Ante este escenario, y considerando el contexto que rodea el compromiso emocional del policía militar en su entorno laboral, el estudio tuvo como objetivo analizar el costo afectivo del trabajo en los policías militares de la región metropolitana de Río de Janeiro.

## MÉTODO

### Diseño del Estudio

Se trata de un estudio transversal, basado en las directrices de Strengthening the Reporting of Observational Studies in Epidemiology (STROBE) para la redacción del manuscrito.

### Lugar del Estudio

La investigación se llevó a cabo en los Batallones de la Policía Militar (BPM) que presentaban los mayores enfrentamientos armados en la Región Metropolitana I del estado de Río de Janeiro, de acuerdo con los altos índices de criminalidad presentados en el Simposio Nacional de Victimización Policial en el Estado de Río de Janeiro, realizado por la Policía Militar en 2019.

### Población y Criterios de Selección

El número de participantes se determinó teniendo en cuenta el total de 44 336 policías militares en el estado de Río de Janeiro. Los criterios de selección fueron ser policía militar, de ambos sexos, de los siguientes rangos jerárquicos: suboficiales (soldados, cabos, sargentos y subtenientes) y oficiales (tenientes, capitanes, mayores, tenientes coroneles y coroneles), distribuidos entre 5 BPM del estado de Río de Janeiro.

Todos los policías de las unidades militares seleccionadas que estaban presentes durante la recopilación de datos fueron considerados para participar, siempre que cumplieran los criterios de aptitud A (sin restricciones de salud) y aptitud B (con algunas restricciones de salud y sin restricción para portar armas) y estuvieran en condiciones de responder a las preguntas de los cuestionarios. Se excluyó a los militares inactivos, de vacaciones o con aptitud C (problemas de orden psíquico u otro problema de salud más grave, con restricción al porte de armas por un tiempo determinado).

### Definición de la Muestra

Para el cálculo de la muestra se utilizó el software EPI-INFO en su versión 7.2.2.16, con un margen de error del 5,0%, un nivel de confianza del 95,0%, una estimación de proporción del 50,0% y una tasa de pérdida del 20,0%. La muestra estratificada fue de n = 417. Sin embargo, otros policías manifestaron su interés en participar en el estudio durante la recopilación de datos y fueron incluidos en la muestra, ya que cumplían los criterios de inclusión. Participaron en el estudio 446 policías militares.

Los batallones se denominaron A, B, C, D y E para garantizar el anonimato, y los participantes se distribuyeron por batallones de la siguiente manera: A (n = 108); B (n = 104); C (n = 98); D (n = 54) y batallón E (n = 82).

### Instrumento de Recopilación de Datos

El instrumento de recopilación consistió en un cuestionario sociodemográfico y la Escala de Evaluación del Costo Humano en el Trabajo (EACHT). El cuestionario, con preguntas sobre el perfil sociodemográfico, de salud y laboral, incluía las siguientes variables: sexo, edad, tener hijos, posición jerárquica, unidad de actuación, tiempo de servicio en la institución militar, condiciones de salud preexistentes, licencia por baja médica y cantidad de horas de sueño. No se realizó ninguna prueba piloto de este instrumento.

La EACHT forma parte del ITRA, inventario validado en Brasil en 2003, adaptado y validado nuevamente en 2004 y 2006, y publicado posteriormente en 2007. El análisis factorial reveló la adecuación de los datos, con un índice de Kaiser-Meyer-Olkin (KMO) = 0,91, lo que indica una excelente adecuación de la muestra para la aplicación del modelo (los valores superiores a 0,80 se consideran muy buenos). El valor propio (eigenvalue) superior a 2 confirma que el factor extraído explica una cantidad significativa de la varianza de los ítems, siendo superior al punto de corte habitual de 1,0. La solución factorial presentó una varianza total explicada del 44,98%, con un 50% de las correlaciones entre los ítems por encima de 0,30, lo que refuerza la consistencia de la estructura factorial.

La EACHT se compone de cinco factores, que analizan las demandas relacionadas con el costo humano en el trabajo: costo físico (preguntas de 1 a 10); costo cognitivo (preguntas de 11 a 20); y costo afectivo (preguntas de 21 a 30)^(10)^. Para cumplir con el objetivo de este artículo, se realizó un recorte de la tesis doctoral de la primera autora, analizando el factor que evalúa el costo afectivo del trabajo^(11)^.

Se trata de una escala tipo Likert de cinco puntos, que indica el grado de exigencia del trabajo, siendo: 1 – nada exigente; 2 – poco exigente; 3 – más o menos exigente; 4 – bastante exigente; y 5 – totalmente exigente. Se realizó un análisis individual de cada ítem del factor costo afectivo de la EACHT mediante la media aritmética y la desviación estándar. El riesgo de enfermedad se evaluó según las orientaciones de los autores, considerando las siguientes medias: por encima de 3,70: evaluación más negativa, riesgo grave de enfermedad; entre 3,69 y 2,30: evaluación moderada, riesgo crítico de enfermedad; y por debajo de 2,29: evaluación más positiva, satisfactoria^(10)^.

### Recopilación de Datos

Inicialmente, se estableció contacto con el Comandante General de la Policía Militar a través de la Coordinación de Asuntos Estratégicos (CAEs), con el fin de explicar la propuesta de la investigación y solicitar autorización para llevarla a cabo en cada unidad. Tras la presentación del documento oficial de aprobación emitido por la CAEs, la investigación se sometió al Comité de Ética de la institución proponente y, tras su aprobación, se inició la recopilación de datos en las diferentes unidades. De este modo, se invitó a los militares a participar en la investigación y se les informó sobre sus objetivos y la importancia de su colaboración.

La recopilación de datos se llevó a cabo entre diciembre de 2021 y junio de 2022, siguiendo un calendario previamente acordado con los participantes, en un lugar definido por los encuestados. Los participantes completaron los instrumentos y se estableció el plazo para su devolución en el siguiente turno. Cuando la devolución no se produjo en el plazo previsto, se realizó un nuevo intento, con lo que se produjo la devolución y no hubo pérdidas.

### Análisis y Tratamiento de los Datos

Para la estructuración y organización de la base de datos, se utilizaron los programas Statistical Package for the Social Sciences (SPSS), versión 13.1. para la plataforma Windows y Excel 365 con el método de doble entrada, con el fin de reducir posibles errores e incongruencias. Todos los análisis se realizaron con un nivel de confiabilidad del 95%, considerando únicamente las respuestas válidas.

Los datos se presentaron en tablas, con sus respectivas frecuencias absolutas y relativas. Las variables cuantitativas (características sociodemográficas y laborales y las del costo efectivo en el trabajo de la EACHT) se presentaron mediante medidas de centralidad y dispersión. Para verificar la relación estadísticamente significativa se utilizaron la prueba de chi cuadrado y la prueba exacta de Fisher y, para evaluar la normalidad de los datos, se optó por la prueba no paramétrica de Kolmogorov-Smirnov en las variables numéricas.

Con el fin de evaluar la diferencia entre tres o más conjuntos de datos que no estaban relacionados, se optó por utilizar la prueba estadística de Kruskal-Wallis.

### Aspectos Éticos

El estudio fue aprobado por el Comité de Ética de la institución proponente, siguiendo las directrices de la Resolución n.º 466/2012, y aprobado por el dictamen n.º 5.137.132. Todos los participantes leyeron el Término de Consentimiento Libre y Esclarecido (TCLE), que fue firmado, confirmando su participación en la investigación, y se entregó a cada participante una copia firmada por el investigador. Cabe destacar que la participación en la investigación fue libre y espontánea, permitiéndose la retirada del consentimiento en cualquier momento, así como la no respuesta a cualquiera de las preguntas, en caso de que el participante no se sintiera cómodo. Por lo tanto, la “n” de cada variable presentó una variación del total de la muestra.

## RESULTADOS

Participaron en la investigación 446 policías militares del estado de Río de Janeiro. Sin embargo, algunas preguntas del formulario no fueron respondidas. Debido a esta pérdida de información, el “n” difiere según las variables.

Del total de participantes, el 86.7% (379) eran hombres y el 13.3% (58) mujeres; en cuanto al estado civil, el 70.8% (311) estaban casados o vivían en pareja, mientras que el 29.2% (128) estaban separados, divorciados, viudos o solteros. En cuanto al número de hijos, el 64,7% (289) afirmaron tenerlos; de ellos, la mayoría (47,4%, 137) tenían solo un hijo.

Se preguntó a los policías militares sobre los motivos de las bajas médicas. En esta pregunta, se podía marcar más de un diagnóstico, por lo que los datos se presentan en valores absolutos, a partir del número de participantes, con los respectivos diagnósticos: crisis de ansiedad (n = 81), depresión (n = 64), síndrome de pánico (n = 21) y trastorno bipolar (n = 17).

Cuando se les preguntó si alguna vez habían pensado en suicidarse, el 15% (66) de los participantes respondió afirmativamente. Cabe destacar que siete participantes no respondieron a la pregunta. En cuanto a los cambios cognitivos, 193 policías respondieron afirmativamente, de los cuales 110 afirmaron haber presentado cambios en la memoria, 63 cambios en la atención y 20 cambios en la percepción.

La Tabla 1 presenta la media, la desviación estándar y la clasificación de riesgo de los ítems que componen el factor coste afectivo del trabajo de la EACHT.

**Tabla 1:** Ítems del factor costo afectivo del trabajo de la Escala de Costo Humano del Trabajo según la media, la desviación estándar y la clasificación de riesgo para los policías militares del estado de Río de Janeiro (n = 446) – Río de Janeiro, Brasil, 2023

Costo Afectivo	Media ± DE*	Clasificación de riesgo
Tener control de las emociones	4,06 ± 1,15	Grave
Tener costo emocional	3,67 ± 1,20	Crítico
Estar obligado a lidiar con agresividad de los demás	3,65 ± 1,33	Crítico
Disfrazar los sentimientos	3,48 ± 1,36	Crítico
Tener que lidiar con órdenes contradictorias	a3,39 ± 1,17	Crítico
Estar obligado a cuidar la apariencia física	3,23 ± 1,32	Crítico
Estar obligado a tener buen humor	2,54 ± 1,32	Crítico
Ser amable con los demás	2,49 ± 1,30	Crítico
Transgredir valores éticos	2,22 ± 1,50	Satisfactorio
Ser sometido a situaciones incómodas	2,17 ± 1,34	Satisfactorio
Estar obligado a elogiar a otros	2,08 ± 1,25	Satisfactorio
Estar obligado a sonreír	1,86 ± 1,20	Satisfactorio

*Desvío Estándar.

Los ítems se presentan en orden decreciente a partir de las medias. Las respuestas más negativas fueron en relación con “Tener control de las emociones” (μ = 4,06 ± 1,15), lo que indica un riesgo grave de enfermedad; y “Tener costo emocional” (μ = 3,67 ± 1,20), con riesgo crítico de enfermedad. La respuesta más positiva fue en relación con “Estar obligado a sonreír” (μ = 1,86 ± 1,20), lo que indica que los policías consideran que no están obligados a sonreír, lo cual es un factor positivo en el contexto laboral. La desviación estándar fue superior a 1,00 en todos los ítems del factor, lo que indica variabilidad en las respuestas.

La Tabla 2 presenta la asociación entre el factor costo afectivo y las variables: ha pensado en el suicidio, motivo de la baja médica, alteraciones cognitivas, edad, tiempo en la PMERJ y horas de sueño al día. Se eligieron las variables que, según la literatura, pueden influir en la relación entre el contexto laboral y el costo humano en el trabajo. Entre las variables analizadas, se observó una asociación estadísticamente significativa entre la evaluación del costo afectivo grave y la baja médica por “trastorno bipolar”, y los cambios cognitivos relacionados con la “percepción” y la “memoria”. También se identificaron diferencias estadísticamente significativas en las tres variables numéricas “Edad”, “Tiempo en la PMERJ” y “Horas de sueño al día”, lo que indica que estas variables estaban asociadas con los niveles de costo afectivo del trabajo entre los policías militares.

**Tabla 2:** Asociación entre el factor costo afectivo y las variables sociodemográficas, laborales y condiciones de salud (n = 446) – Río de Janeiro, Brasil, 2023.

	EACHT – Costo Afectivo		
Variables	Grave n (%)	Crítico n (%)	Satisfactorio n (%)	p-valor
**¿Alguna vez ha pensado en suicidarse?**				
Si	26 (40,0)	25 (38,5)	14 (21,5)	0,568**
No	122 (33,6)	162 (44,6)	79 (21,8)
**Motivo de Licencia Médica**				
Depresión	25 (39,1)	22 (34,3)	17 (26,6)	0,252**
Crisis de ansiedad	31 (38,3)	33 (40,7)	17 (21,0)	0,706**
Trastorno bipolar	**9 (56,2)**	2 (12,5)	5 (31,3)	0,035**
**Alteraciones cognitivas**				
Percepción	**13 (65,0)**	6 (30,0)	1 (5,0)	0,009**
Atención	29 (46,0)	26 (41,3)	8 (12,7)	0,053**
Memoria	**48 (43,6)**	47 (42,8)	15 (13,6)	0,015**
	**Mediana (P_25_; P_75_)**	**Mediana (P_25_; P_75_)**	**Mediana (P_25_; P_75_)**
Edad	37,0 (33,0; 41,0)	37,0 (33,0; 42,0)	41,0 (36,0; 46,0)	**< 0,001^A^**
Tiempo en la PMERJ**	9,0 (3,0; 14,5)	9,0 (3,0; 18,3)	12,5 (8,0; 21,3)	**< 0,001^A^**
Horas de sueño por día	6,0 (5,5; 7,0)	6,0 (6,0; 7,0)	7,0 (6,0; 8,0)	**0,034^A^**

*Chi-cuadrado.

**Policía Militar do Estado do Río de Janeiro.

^A^Kuskal-Wallis

## DISCUSIÓN

Los resultados de esta tesis corroboran estudios anteriores que indican el predominio de policías militares varones, con edades comprendidas entre los 30 y los 40 años, casados o en unión libre, y con uno o dos hijos. Este perfil demográfico pone de manifiesto la importancia del contexto conyugal y familiar para comprender los impactos de la actividad profesional en la vida personal de estos trabajadores^(11,12)^.

En este sentido, estas características influyen directamente en las decisiones reproductivas, que han revelado una tendencia a la reducción del número de hijos entre los policías militares, fenómeno frecuentemente asociado a las especificidades de la profesión. Además, el aumento de la mortalidad entre los policías en el estado de Río de Janeiro, con un crecimiento del 45,5% en 2021, lo que suma un total de 64 muertes, intensifica el temor de dejar huérfanos a los hijos, lo que repercute negativamente en la planificación familiar^(13)^.

Estos aspectos, sumados al elevado costo afectivo de la profesión, se manifiestan en síntomas como ansiedad, miedo y depresión, lo que compromete tanto la salud mental como las relaciones interpersonales de estos profesionales^(14)^.

El entorno laboral ejerce una influencia significativa en la motivación de los profesionales y en el riesgo de enfermar, como se ha demostrado en estudios con diferentes categorías laborales, como policías militares, profesionales de la salud y la educación. Las jornadas laborales excesivas, las condiciones inadecuadas y la falta de formación adecuada son factores comunes que contribuyen a problemas de salud física y mental, aunque la intensidad y la repercusión de estos factores varían según el sector de actividad. La sobrecarga mental y emocional puede deberse a jornadas prolongadas, la casi total ausencia de descansos, la alta responsabilidad, las limitaciones técnicas y materiales, la elevada demanda y, a menudo, la baja remuneración. Estas condiciones, junto con las presiones organizativas, pueden generar un impacto negativo en la relación de los profesionales con los usuarios y aumentar el riesgo de enfermedad, lo que exige un análisis cuidadoso de las especificidades de cada entorno laboral para el desarrollo de estrategias eficaces de prevención y promoción de la salud^(15-17)^.

En esta investigación, los principales motivos de baja médica reflejan el sufrimiento psíquico de la categoría y tienen como consecuencia las patologías. En este caso, la capacidad de la organización del trabajo militar de debilitar a los individuos y propiciar desequilibrios psicológicos, cuando el trabajador ha agotado todos los recursos de origen psicoafectivo e intelectual para responder a las exigencias del trabajo^(18)^. Esta realidad no se limita a los policías militares, ya que los datos de la Seguridad Social de Brasil confirman un aumento del 29% en los años 2019 y 2020 en la concesión de prestaciones por enfermedad, destacando las enfermedades relacionadas con trastornos mentales y conductuales, que son la tercera causa de bajas laborales por incapacidad en Brasil^(19)^.

Específicamente en relación con el trabajo de los policías militares, el contacto directo o indirecto con eventos violentos que resultan en muerte o amenaza de muerte, lesiones graves y violación sexual conlleva experiencias traumáticas para los involucrados, que, a su vez, pueden desencadenar diversas enfermedades psicoemocionales y otras afecciones, sobre todo el trastorno de estrés postraumático (TEPT), depresión, abuso de sustancias, problemas sociales, sentimiento de frustración con el trabajo y aumento de la demanda de servicios médicos^(20)^.

El costo afectivo en el trabajo se caracteriza por la demanda emocional en respuesta a reacciones afectivas, sentimientos y estado de ánimo^(10)^. En la evaluación de los policías militares de este estudio, el costo afectivo del trabajo que realizan ofrece un riesgo crítico de enfermedad, destacándose entre los ítems evaluados más negativamente el control de las emociones, el costo emocional y la obligación de lidiar con la agresividad de los demás, siendo el primero evaluado como grave. En el contexto laboral de los policías militares, es evidente que, además de enfrentarse a diversas exigencias en el entorno laboral, la violencia forma parte de su día a día, siendo uno de los motivos por los que hay una gran manifestación de emociones en el desempeño de sus funciones.

En medio de este contexto caótico, la aceptación de la violencia en las ciudades se percibe a través de un proceso repetitivo, que se manifiesta en las prácticas policiales arraigadas a lo largo de los años. Además, es importante destacar la importancia de abordar el problema desde el sentimiento de miedo, que desempeña un papel fundamental en el aumento de la violencia^(21)^. En esta lógica, el agente de seguridad pública ocupa una posición de enlace causante de la violencia del Estado, invirtiendo su desempeño profesional real en función de lo que pueda derivarse de su (re)acción basada en una actitud violenta originada en el temor, ocasionado por la adversidad del contexto social, como las condiciones de trabajo, la capacitación, el acompañamiento psicológico y la adecuación de la carga horaria.

Abstenerse ante este escenario implica un grave peligro para la sociedad. Esto se debe a que ignorarlo es dar pie a que un policía con problemas psicológicos pueda, en momentos de tensión, cometer abusos y actuar de forma agresiva. De hecho, los trastornos mentales debilitan la capacidad de razonamiento de estos profesionales, ya que afectan a su capacidad para utilizar recursos que permitan encontrar soluciones que no impliquen la agresión y la violencia presentes en el día a día de la profesión^(22)^.

Desde la perspectiva del costo afectivo en el trabajo, la actuación policial en las calles implica lidiar con diversas demandas emocionales, como interactuar con infractores, delincuentes, víctimas y la comunidad en general, lo que requiere la habilidad de expresar diferentes sentimientos. La identidad profesional es esencial en este escenario, ya que permite al policía adoptar estrategias para expresar emociones según la situación.

Sin embargo, es importante destacar que los policías tratan a diario con públicos diferentes, cada uno con necesidades emocionales específicas, lo que hace que el control emocional sea aún más difícil, ya que deben mostrar empatía y solidaridad con las víctimas, autoridad y firmeza con los sospechosos y delincuentes, y profesionalidad al tratar con la población en general^(23)^. Esto nos lleva a pensar que una de las responsabilidades más importantes y desafiantes de la policía es mantener la violencia bajo control sin dejarse afectar por ella.

Por lo tanto, se deben considerar los efectos simbólicos, prácticos y políticos tanto en la esfera social como en el día a día del profesional de la Seguridad Pública, que necesita desarrollar estrategias tanto físicas como psicológicas para lidiar con la realidad de su actividad, que implica violencia dirigida haciasí mismo y hacia los demás^(20)^. Esta actividad interfiere en la vida privada, el rigor militar y la exposición a la violencia moldean la forma de relacionarse. Esta influencia va más allá de las relaciones, pasando a orientar el carácter del profesional, mostrando así la dimensión que la profesión puede tener en la vida del policía^(24)^.

La falta de apoyo institucional para hacer frente a la violencia laboral, asociada al riesgo inminente de muerte y a las crecientes presiones organizativas, configura un escenario propicio para el sufrimiento psicológico de los policías militares. Esta condición, sumada al desgaste físico y la vulnerabilidad emocional, puede desencadenar respuestas conductuales inadecuadas durante situaciones críticas y operaciones de alto riesgo^(25)^.

En este sentido, un estudio realizado con policías militares de Minas Gerais reveló que el 14,8% de los participantes presentaban síntomas significativos de depresión y agotamiento. Estos cuadros de enfermedad emocional estuvieron fuertemente asociados a factores como el estrés laboral, la insatisfacción con el trabajo, la percepción negativa de la salud mental y la baja resiliencia, lo que pone de manifiesto el impacto negativo de las condiciones laborales y las exigencias institucionales y sociales sobre la salud mental de estos profesionales^(26)^.

Aunque no existe una relación estadística significativa, el 15% de los policías ha pensado en el suicidio. En este sentido, una investigación realizada en una institución policial-militar del sur de Brasil identificó 14 casos entre los años 2012 y 2016^(27)^. El gran cansancio que experimentan a diario los policías en su entorno profesional es el principal factor que conduce al deterioro tanto mental como físico, lo que puede llevar al suicidio como una forma de poner fin a su propio tormento^(28)^.

En consonancia con estos resultados, una revisión integradora que buscó identificar los temas más relevantes relacionados con la salud mental de los policías en la literatura, en el período de 2012 a 2018, permitió contextualizar los estudios sobre este tema. Quedó claro que, a pesar de haberse realizado en diferentes países, las investigaciones señalan el estrés, el TEPT, la depresión, la ansiedad, el agotamiento profesional y el suicidio como los principales problemas de salud estudiados^(29)^. En este sentido, el TEPT se relaciona como una de las principales causas que conducen al suicidio, lo que se explica por la exposición constante a eventos traumáticos durante el cumplimiento de sus obligaciones^(30)^.

La búsqueda de ayuda de profesionales especializados es crucial para identificar señales y factores que puedan indicar trastornos mentales, evitando acciones drásticas o la negación de la existencia del problema. La falta de apoyo en materia de salud mental es alarmante, especialmente ante los cambios en las relaciones sociales, que pueden aumentar la presión y desencadenar crisis^(31)^.

Aunque los policías militares se enfrentan a rutinas de alta exposición a riesgos, violencia y estrés intenso, el acceso constante a las armas refuerza una cultura institucional de invulnerabilidad, en la que expresar fragilidad psicológica representa una amenaza para la carrera. Esta configuración intensifica el estrés laboral y dificulta la comunicación de los síntomas psiquiátricos: muchos optan por ocultarlos por temor a dañar su reputación, a represalias o a la falta de confidencialidad. La rigidez jerárquica y la ausencia de espacios institucionales de diálogo agravan esta negación del sufrimiento emocional, reduciendo el uso de los servicios de salud mental e impidiendo el acceso efectivo al tratamiento, lo que perpetúa un ciclo de enfermedades no diagnosticadas o tratadas formalmente^(32)^.

La vida cotidiana de estos profesionales, según una revisión integradora publicada en 2024, evidencia que la profesión policial es una de las más estresantes, con una alta incidencia de enfermedades psíquicas relacionadas con el trabajo^(25)^. Factores como la jerarquía rígida, la ausencia de días libres, los turnos irregulares, la presión operativa y las exigencias emocionales crónicas hacen que estos trabajadores sean vulnerables a las enfermedades mentales^(32)^. Los estudios también señalan que las bajas por trastornos psiquiátricos (como depresión, ansiedad y estrés postraumático) son recurrentes, lo que refuerza la importancia de las políticas institucionales de atención^(25)^. Estos elementos ponen de relieve la urgencia de intervenciones capaces de romper el ciclo de silencio emocional que compromete la salud mental de estos profesionales.

Los resultados indican que el costo afectivo que enfrentan los policías militares está asociado con enfermedades emocionales, incluyendo trastornos del estado de ánimo, alteraciones cognitivas relacionadas con la percepción y la memoria, además de dificultades para dormir. El entorno de trabajo, especialmente cuando el cuartel está situado en zonas con alta incidencia de enfrentamientos armados, impone intensas demandas emocionales y contribuye al desarrollo de estos cuadros clínicos. Estas condiciones afectan significativamente la salud mental de los profesionales, lo que se refleja en licencias médicas por trastornos como el trastorno bipolar, deterioro cognitivo y variaciones relacionadas con la edad y el tiempo de servicio^(25)^.

En esta investigación, se observó que los policías más jóvenes y con menos tiempo de servicio informaron que el costo afectivo del trabajo era grave o crítico, lo que indica una mayor vulnerabilidad psicosocial. Estos resultados difieren de los observados en otro estudio, realizado con policías militares de Sergipe, que asoció la mala calidad del sueño y el deterioro de la calidad de vida principalmente a los profesionales más jóvenes, con una media de 40 años y hasta siete años de servicio^(33)^. Este trabajo identificó impactos en las dimensiones biológica, psicológica, relacional y económica, revelando que la privación del sueño compromete sustancialmente la salud mental de estos profesionales. Las divergencias entre los estudios pueden reflejar particularidades regionales y operativas de las corporaciones, lo que refuerza la importancia de considerar estas variables al analizar las enfermedades en el trabajo policial.

Aunque las condiciones de trabajo en el servicio policial militar son exigentes y a menudo agotadoras, ciertos componentes del costo afectivo del trabajo se perciben de manera menos negativa. En un análisis completo, aspectos como no sentirse obligado a sonreír o elogiar a las personas obtuvieron las puntuaciones medias más bajas, lo que indica que estas exigencias emocionales se consideran menos penosas. Además, muchos policías reportan un fuerte sentido de pertenencia a la corporación, orgullo institucional y satisfacción al realizar tareas punitivas en nombre del Estado, lo que sugiere una dimensión emocional positiva incluso ante las adversidades y el estrés continuo^(6)^. Estos hallazgos coinciden con este estudio y muestran que, incluso en entornos con altos niveles de estrés laboral, muchos policías militares presentan niveles medios a altos de compromiso con el trabajo, destacando el entusiasmo, la dedicación y el orgullo en la función métrica, lo que refuerza que ciertos aspectos afectivos del trabajo pueden generar resiliencia emocional, en lugar de un sufrimiento significativo.

Los resultados de esta investigación confirman que la realización de tareas externas durante las rondas ostensivas y el enfrentamiento directo con la delincuencia son indicadores significativos de agotamiento emocional entre los policías. Estas actividades exponen a los policías a altos niveles de violencia y riesgo, lo que contribuye a un estrés considerable. Además, estas funciones se realizan a menudo en condiciones laborales precarias, lo que exacerba el impacto negativo en el bienestar de los policías.

Los problemas afectivos en este entorno laboral son alarmantes y se han vuelto más frecuentes. Factores como el estrés, la presión por obtener resultados, la competencia feroz y el desequilibrio entre la vida profesional y personal pueden estar contribuyendo a la aparición de problemas emocionales, incluidos los identificados en esta investigación, como la ansiedad, la depresión y el trastorno bipolar.

Como limitación de la investigación, se puede considerar el tipo de estudio transversal, que no permite afirmar una relación de causa y efecto entre las variables investigadas. Por lo tanto, se sugiere el diseño de estudios longitudinales para comprender el fenómeno a lo largo del tiempo.

El estudio contribuyó a la reflexión sobre la importancia de la prevención de problemas mentales en el lugar de trabajo, lo que exige la colaboración entre las organizaciones y los trabajadores. Es esencial promover un entorno saludable, concienciar sobre la importancia de la salud mental y buscar ayuda profesional cuando sea necesario. Garantizar la salud mental de los policías es fundamental para su felicidad y calidad de vida, lo que corrobora la urgente necesidad de intervenciones y apoyos continuos en esta área.

## CONCLUSIÓN

El análisis del costo afectivo del trabajo de los policías militares de la región metropolitana de Río de Janeiro obtuvo una evaluación crítica para la mayoría de los factores presentados, siendo la respuesta más negativa, con una evaluación de riesgo grave de enfermedad, la relativa al control de las emociones, seguida de la del costo emocional, con riesgo crítico de enfermedad.

El análisis de asociación indicó que la evaluación del costo afectivo grave estaba relacionada con la baja médica por trastorno bipolar y alteraciones cognitivas de la percepción y la memoria. También se observó una asociación entre la edad, el tiempo de trabajo en la policía militar y las horas de sueño, lo que indica que los policías más jóvenes, con menos tiempo de trabajo y menos horas de sueño, presentaron una evaluación grave del costo afectivo en el trabajo. Otro dato que merece ser destacado es el hecho de que el 15% de los policías han pensado en el suicidio. La naturaleza del trabajo del policía militar expone a este profesional al desgaste emocional, a problemas afectivos y a alteraciones del estado de ánimo, que pueden tener como consecuencia problemas psiquiátricos y alteraciones cognitivas, lo que conduce a enfermedades, bajas e incluso a pensamientos suicidas.

## Data Availability

El conjunto de datos que respaldan las conclusiones de este estudio está disponible previa solicitud al autor correspondiente.
